# Modelling survival: exposure pattern, species sensitivity and uncertainty

**DOI:** 10.1038/srep29178

**Published:** 2016-07-06

**Authors:** Roman Ashauer, Carlo Albert, Starrlight Augustine, Nina Cedergreen, Sandrine Charles, Virginie Ducrot, Andreas Focks, Faten Gabsi, André Gergs, Benoit Goussen, Tjalling Jager, Nynke I. Kramer, Anna-Maija Nyman, Veronique Poulsen, Stefan Reichenberger, Ralf B. Schäfer, Paul J. Van den Brink, Karin Veltman, Sören Vogel, Elke I. Zimmer, Thomas G. Preuss

**Affiliations:** 1 Environment Department, University of York, Heslington, York YO10 5NG, United Kingdom; 2 Swiss Federal Institute of Aquatic Science and Technology, 8600 Dübendorf, Überlandstrasse 133, Switzerland; 3 Akvaplan-niva, Fram – High North Research Centre for Climate and the Environment, 9296 Tromsø, Norway; 4 Department of Plant and Environmental Science, University of Copenhagen, Thorvaldsensvej 40, 1871 Frederiksberg, Denmark; 5 Univ Lyon, Université Lyon 1, UMR CNRS 5558, Laboratoire de Biométrie et Biologie Évolutive, F-69100 Villeurbanne, France; 6 Bayer CropScience Aktiengesellschaft, BCS AG-R&D-D-EnSa-ETX-AQ, Monheim, Deutschland; 7 Alterra, Wageningen University and Research centre, P.O. Box 47, 6700 AA, The Netherlands; 8 RIFCON GmbH, Goldbeckstraße 13, 69493 Hirschberg, Germany; 9 Research Institute for Ecosystem Analysis and Assessment (gaiac), Kackertstrasse 10, 52072, Aachen, Germany; 10 Safety and Environmental Assurance Centre, Colworth Science Park, Unilever, Sharnbrook, Bedfordshire, United Kingdom; 11 DEBtox Research, De Bilt, The Netherlands; 12 Utrecht University, Institute for Risk Assessment Sciences (IRAS), 3584 Utrecht, Netherlands; 13 European Chemicals Agency, Annankatu 18, FI-00121, Helsinki, Finland; 14 French Agency for Food, Environmental and Occupational Health Safety (ANSES), Regulated Product Assessment Directorate, 14 rue Pierre et Marie Curie 94704 Maisons Alfort, France; 15 Dr. Knoell Consult GmbH, Dynamostr. 19, 68165 Mannheim, Germany; 16 Institute for Environmental Sciences, University Koblenz-Landau, Fortstraße 7, 76829 Landau, Germany; 17 Department of Aquatic Ecology and Water Quality Management, Wageningen University, Wageningen University and Research centre, P.O. Box 47, 6700 AA, The Netherlands; 18 Department of Environmental Health Sciences, University of Michigan, Ann Arbor, Michigan 48109-2029, USA; 19 Ibacon GmbH, Arheilger Weg 17, 64380 Roßdorf, Germany; 20 Bayer CropScience Aktiengesellschaft, BCS AG-R&D-D-EnSa-Emod, Monheim, Germany

## Abstract

The General Unified Threshold model for Survival (GUTS) integrates previously published toxicokinetic-toxicodynamic models and estimates survival with explicitly defined assumptions. Importantly, GUTS accounts for time-variable exposure to the stressor. We performed three studies to test the ability of GUTS to predict survival of aquatic organisms across different pesticide exposure patterns, time scales and species. Firstly, using synthetic data, we identified experimental data requirements which allow for the estimation of all parameters of the GUTS proper model. Secondly, we assessed how well GUTS, calibrated with short-term survival data of *Gammarus pulex* exposed to four pesticides, can forecast effects of longer-term pulsed exposures. Thirdly, we tested the ability of GUTS to estimate 14-day median effect concentrations of malathion for a range of species and use these estimates to build species sensitivity distributions for different exposure patterns. We find that GUTS adequately predicts survival across exposure patterns that vary over time. When toxicity is assessed for time-variable concentrations species may differ in their responses depending on the exposure profile. This can result in different species sensitivity rankings and safe levels. The interplay of exposure pattern and species sensitivity deserves systematic investigation in order to better understand how organisms respond to stress, including humans.

## Introduction

### The General Unified Threshold model of Survival (GUTS)

Effects of stressors on survival over time of systems, such as organisms or mechanical systems, are studied in a wide range of disciplines, such as toxicology^1^,^2^,^3^,^4^, biology^5^,^6^,^7^, medicine^8^,^9^,^10^, engineering^11^,^12^ and social sciences^13^,^14^. In this paper, we take an ecotoxicological perspective, where we assess the potential of a specific application of survival analyses, namely as a module in toxicokinetic-toxicodynamic (TK-TD) modeling. We note, however, that similar survival analyses could be carried out for engineering or social science problems^13^,^14^. The commonly asked question in survival analysis is how one or multiple stressors affect survival over time and how we could predict it. Any answer to these questions entails specifying where the stressor is quantified (e.g. inside or outside the organism), deciding how to model compensatory processes (e.g. damage repair) and whether death of an organism or failure of a system is best viewed as a stochastic or as a deterministic event.

TK-TD models quantify the time-course of internal concentrations of chemicals, which is defined by uptake and elimination rates of chemicals (toxicokinetics), as well as the processes leading to toxic effects (toxicodynamics), which include damage accrual and recovery, as well as the death mechanisms explained below. Many TK-TD models exist in literature, each having their own (often implicit) assumptions of damage and mortality processes^3^,^15^. The General Unified Threshold model of Survival^3^ (GUTS) provides a theoretical framework for deriving consistent model equations for different choices and assumptions about stressor quantification, compensatory processes and the nature of the death process. Because GUTS unifies TK-TD assumptions, it allows researchers to systematically investigate how well different model assumptions perform when it comes to predicting survival of organisms. Moreover, due to its general nature and the importance of survival analysis for different fields of science, GUTS has diverse application domains.

GUTS originated in an ecotoxicology context, which is why the dose metric of a stressor in GUTS models is typically the concentration of a toxicant in the medium surrounding an organism (e.g., a concentration in a water body), the concentration of a toxicant inside the organism (i.e. internal concentration), or the damage caused by the toxicant. GUTS equations link a dose metric to survival, but GUTS does not prescribe any specific dose metric. Rather, GUTS offers a framework to use different dose metrics (i.e. external concentration of a toxicant, internal concentration of a toxicant, damage or scaled damage) in a consistent way. The decision on which dose metric to use depends on the research question and available data. Subsequently, GUTS provides the equations to estimate mortality when the dose metric exceeds a threshold^3^,^16^. One can assume that i) the threshold is distributed within a population, and when exceeded, the individual dies (individual tolerance, IT, assumption), or ii) there is one common threshold for all individuals, and when exceeded, the probability of an individual to die increases (stochastic death, SD, assumption)^3^,^17^,^18^. The detailed implications of these assumptions are discussed elsewhere^3^,^19^,^20^. The GUTS framework provides a unification of the abovementioned TK-TD model approaches by assuming that the threshold is distributed within a population and, when exceeded, the probability of an organism dying increases. This is the GUTS proper model. From that model, two simpler model implementations can be derived, namely GUTS-IT and GUTS-SD^3^,^16^. All three models, GUTS proper, GUTS-IT and GUTS-SD, can be applied, but because GUTS-IT and -SD have less parameters than the GUTS proper model, they are more easily calibrated and applied. Table 1 lists the various GUTS model implementations and notes what dose metric is used, what parameters need to be estimated, and the typical cases where one would use each specific implementation of the model. Equations of the GUTS proper model can be found in the original publication^3^. Equations for GUTS-SD and GUTS-IT have also been previously published^16^,^21^,^22^,^23^, but they are also reported in the Supplementary Information (SI) for clarity.

### GUTS proper versus special cases GUTS-SD and GUTS-IT

The GUTS proper model integrates both death mechanisms SD and IT into a single framework. In practice, however, it is usually the special cases of GUTS-SD or GUTS-IT that are used in ecotoxicology. They are easier to implement into software, faster to run (although speed is becoming less of an issue with efficient algorithms, such as in the GUTS R-package, see SI), and place less stringent requirements on the dataset used for calibration. Choosing between SD and IT is difficult. Theoretical reasons to prefer one over the other are lacking and in practice they often provide a similar goodness-of-fit to the data^20^, present work. Even in the cases where either GUTS-IT or GUTS-SD clearly gives a better fit to measured data, this better fit may simply be because a model assumption is not met in a particular data set. To illustrate, if SD is the ‘actual’ mechanism, an unrecognized decrease in external concentration (or in bioavailability) over time may easily lead to a survival pattern more akin to IT (mortality levelling-off in time). For some research questions, it would be best to use the GUTS proper model (which combines SD and IT) and observe to what extent both mechanisms can help interpret the data. However, compared to the limit cases of SD or IT, the GUTS proper model requires estimation of one additional parameter. In practice, survival datasets used in ecotoxicology generally do not contain enough information to estimate all parameters of the GUTS proper model with sufficient precision. This is because a large number of individuals is required to provide strong information on probabilistic events and because the choices of concentrations and exposure durations also matter^23^. It is not clear which experimental designs can actually identify both death mechanisms and to what degree of precision.

### GUTS in ecotoxicology and environmental risk assessment of chemicals

Here, we assess to what extent we can use GUTS to answer environmental risk assessment questions^24^,^25^,^26^. Toxic effects are usually determined in laboratory tests using individual organisms of standard species while keeping environmental and chemical exposure conditions as constant as possible. Extrapolating from standard toxicity test results to natural conditions where large numbers of species are exposed to fluctuating concentrations of numerous chemicals raises a range of issues, including variability in species sensitivity, interactions between species in communities and variabilities in exposure concentration and duration. GUTS provides a means of extrapolating laboratory test results to environmental conditions, but such extrapolation requires robust calibration and reliable forecasting.

We investigate calibration and forecasting of survival with GUTS in three case studies. In the first study, we discuss parameter identification for GUTS proper with respect to experimental design. To identify the data requirements for a precise and accurate GUTS proper parameterization, we use synthetic concentration-survival datasets. In the second study, we investigate the potential benefits of using GUTS for toxicity extrapolation across exposure patterns and time scales. We assess the predictive ability of GUTS by forecasting survival across different exposure patterns and quantifying uncertainty. Specifically, we assessed how well GUTS model parameters, calibrated with short-term survival data of *Gammarus pulex* exposed to pesticides, forecast effects of longer-term pulsed exposures. In the third study, we test the ability of GUTS to estimate 14-day lethal effect concentrations (LC_50_) of malathion for a range of species and use these estimates to build a species sensitivity distribution (SSD). In so doing, we demonstrate how using GUTS may help our understanding of the interplay between exposure pattern and species sensitivity under time-varying concentrations of chemicals.

## Study 1: Optimizing experimental design for calibration of the GUTS proper model

To understand what data are needed to ensure a robust calibration of GUTS proper, synthetic data sets were created for *G. pulex* exposed to malathion, with different numbers of individuals per test concentration, different test durations and different numbers of test concentrations. This synthetic data was used to calibrate GUTS proper. Best fit values with 95% credible intervals for GUTS proper parameters were then estimated and compared.

### Methods

In creating synthetic survival data, there are many degrees of freedom regarding the choice of observation times, concentrations and exposure scenarios (e.g., constant or time-varying). We decided to restrict our synthetic data to one particular case study where *G. pulex* are exposed to eight concentrations of malathion in water (0, 0.345–3.837 nmol/L, 20 individuals/concentration) for four days^27^ (see Table S1 in SI). Scaled internal concentration (SIC, Table 1) was chosen as a dose metric in this example. The change in scaled internal concentration of malathion in *G. pulex* (C*_i_) over time is given by dC*_i_(t)/dt = *k*_*e*_(*C*_*w*_(t) − C*_i_(t), where *Cw* is the concentration of malathion in water in nmol/L and *k*_*e*_ is the dominant rate constant. This is a constant that represents a combination of several physiological processes (e.g. elimination rate, damage repair rate; units: d^−1^). We started by fixing *k*_*e*_ = 1 d^−1^, which provided a situation where 98% of the toxicokinetic and toxicodynamic steady state is reached after four days (i.e. the scaled internal concentration reaches a plateau). Because our focus was on the experimental conditions needed to allow a calibration of the GUTS proper model, we wanted to ensure that this parameter can, in principle, be estimated properly from the simulated data (see discussion in SI). Similarly, we wanted to have a situation where both death mechanisms (IT and SD) play a substantial role in explaining observed gammarid mortality over time. We achieved this by fixing the (log-normal) distribution of the threshold *z*, such that 95% of the individuals are within a factor 2 from the median threshold (*F*_*s*_ = 2, see SI). The remaining parameters were estimated by fitting GUTS proper to the original malathion survival data (for the resulting parameter fits, see Table S18 in SI). Using the resulting parameter set, we generated synthetic data for different experimental setups that vary in test duration (4 or 10 days), number of animals per treatment (20 or 100) and number of exposure treatments (5 or 8). For each setup, we generated three replicate datasets. Replicate datasets differed from each other because of the stochastic nature of the death mechanism, i.e., the choice of the threshold for each individual from a frequency distribution and the hazard rate that produces a probability to die at each time point. No additional variability was introduced; hence the model parameters themselves were kept constant for each individual to the values in Table S18 in SI. GUTS proper was fitted to the synthetic data sets with Bayesian methods using the R statistical software and with a likelihood method using Matlab (see SI for details about model software packages and code).

### Results

**Figure 1:**
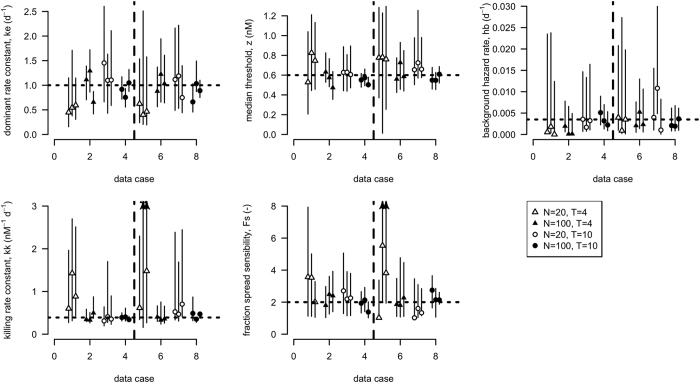
Parameter estimates; best-fit values with 95% credible intervals, resulting from the fits on the synthetic data. Dotted horizontal line indicates the true parameter value that was used to produce the synthetic data sets. Data cases have bioassay designs which differ in number of animals (N), number of days test duration (T). Vertical broken line separates the datasets with 8 exposure concentrations (left) from those with only 5 (right) exposure concentrations. Arrows on confidence intervals indicate that the error bar extends much further (truncated to improve readability).

The results of the parameter identifiability analysis using the Bayesian approach are shown in Fig. 1. Results from the likelihood approach are provided in the SI and are very similar to the results using the Bayesian approach. The data with 100 animals per treatment allowed parameter estimation of the GUTS proper model with reasonable accuracy and precision (see error bars in Fig. 1). For the datasets with 20 animals, the precision and accuracy were much lower for some parameters, including the killing rate constant *k*_*k*_ and median threshold *z*. Increasing the number of test animals or the duration of the experiment substantially reduced the uncertainty around the median value of *z*. In several cases, the confidence interval included one of the two limit cases (GUTS-SD or GUTS-IT), as indicated by intervals for *F*_*s*_ that included 1 (GUTS-SD) and intervals for *k*_*k*_ that went to infinity (GUTS-IT). With a large number of test animals, *k*_*k*_ is better estimated, but the spread of sensitivities (*F*_*s*_) is still uncertain in several cases. This is consistent with datasets often being well described by both GUTS-IT and GUTS-SD^20^.

### Discussion

**Figure 2:**
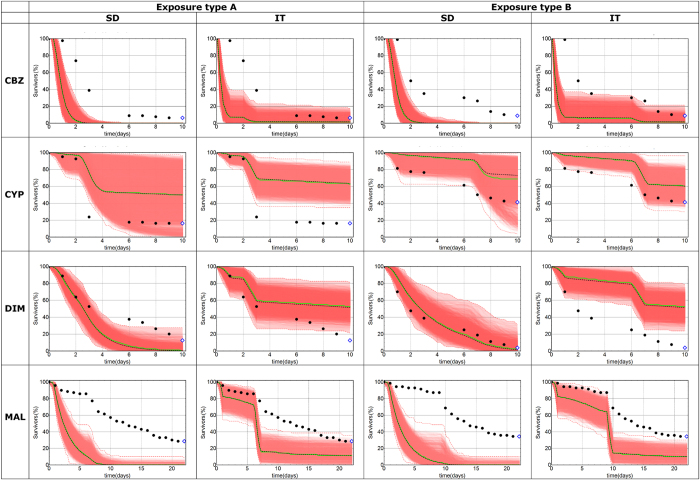
Forecasted and observed survival. The shaded areas indicate the confidence regions (95% parametric uncertainty, 100% stochasticity, 10000 simulations), while the solid green line is the median of these predictions. The more intense the red, the more predictions are overlapping. The observed survival is shown with black dots, but the last data points are highlighted with blue diamonds because those are also shown in Fig. 3.

**Figure 3:**
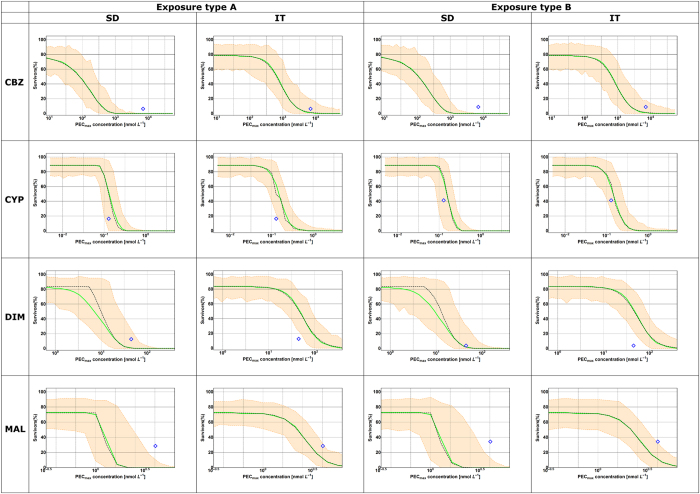
Forecasted dose response curves at the last day of the repeated pulsed exposure experiments. The shaded areas indicate the confidence regions (95% parametric uncertainty, 100% stochasticity, 10000 simulations), while the solid green line is the median of these predictions and the observed survival is shown as blue diamond.

From Fig. 1, we evaluated the precision and accuracy with which each parameter of GUTS proper can be estimated. Even when parameter estimates have a very large uncertainty, the model predictions can still be sufficiently reliable for practical purposes because the correlation structure of the parameter estimates and the structure of the model constrain the model predictions (Figs 2, 3 and S1–4 in SI). The typically observed strong correlation among GUTS parameters means that the confidence regions around model predictions are not as wide as insinuated by the individual parameter confidence intervals.

Estimating the parameter values for the GUTS proper model with a reasonable precision requires more complete toxicity datasets than those currently collected from the standardized ecotoxicity tests used in environmental risk assessment applications. Synthetic data provide insight into the usefulness of various experimental designs for estimating GUTS proper model parameters, and help optimizing these designs for different purposes and under different constraints (Fig. 1). More research is needed to identify how a given number of test organisms is best split between treatments, i.e. how we can best divide a certain number of available test organisms over treatment groups and how we select exposure concentrations and observation times to collect the optimal amount of information for the calibration of the GUTS proper model.

Prior to an ecotoxicity test, the expected gain of knowledge about model parameters can be used as an objective function to develop optimal test designs^23^. However, what is optimal will depend on which type of information is required. For example, the optimal design to estimate 4-day median lethal concentration (LC_50_) values will differ from the design needed to estimate *k*_*e*_, and from the design needed to minimize the uncertainty in predicted mortality in response to a given exposure scenario. Furthermore, optimal design depends on the properties of the chemical and species. For example, for chemicals with ‘slow’ kinetics (i.e. small values of elimination, recovery or dominant rate constants), we need longer test durations to estimate all model parameters precisely. By contrast, for chemicals with ‘fast’ kinetics, we can use shorter test durations, but may require more observations over a single day^28^. Investigating synthetic data allows us to explore the potential parameter space and derive general principles for optimal experimental design.

## Study 2: Forecasting survival across different exposure patterns

The aim of this study was to assess the ability of GUTS to forecast survival of organisms exposed to varying patterns of exposure. Therefore, GUTS -SD and GUTS- IT (Table 1) were calibrated with survival data for *G. pulex* exposed constantly to pesticides for four days. These two model implementations were then used to simulate effects of longer-term pulsed exposures. Finally, the simulated survival rates were compared to experimental data from a repeated pulse exposure ecotoxicity test (Tables S1–S12 in SI). We studied four pesticides from three different classes: the benzimidazole fungicide carbendazim, the pyrethroid insecticide cypermethrin, and the organophosphate insecticides dimethoate and malathion. Scaled internal concentration (SIC) was chosen as a dose metric in this example again for simplicity and because this option was found to perform equally well as the more complex approach based upon scaled internal damage for the purpose of extrapolating over concentration and time^16^.

### Methods

For the model calibration, unexposed animals (i.e. controls) from the acute toxicity tests (i.e. four day constant exposure, Tables S1–S4 in SI) were used to estimate the background hazard rate (*h*_*b*_(t)). Next, GUTS -IT and GUTS -SD were separately fit to survival data from acute toxicity tests with *G. pulex* (constant exposure to malathion, dimethoate, cypermethrin and carbendazim). GUTS -SD or GUTS -IT parameters values were obtained by maximum likelihood estimation^3^. Single parameter confidence intervals (Table S14 in SI) were approximated by likelihood profiling (critical value from the Chi-square distribution, df = 1, α = 0.05)^29^,^30^. To approximate the joint confidence regions of all parameters, we selected those parameter sets from the optimization procedure (simulated annealing) that were not rejected in a likelihood ratio test (critical value from Chi-square distribution, df = 3, α = 0.05). These parameter sets were subsequently used to generate forward predictions and uncertainty intervals. For the forecasting of survival across pulsed exposures, the control survival in the pulse ecotoxicity tests was used.

Subsequently, for the four chemicals, survival of *G. pulex* was simulated over 10 days (or 28 days in the case of malathion exposure) and for two different pulsed-exposure regimes and compared with survival observed in independent experiments (see SI for experimental details and data). The pulsed-exposure regimes A and B consisted of two one-day exposures with a short (A) or longer (B) interval in between exposures (left panel in Figs S1–S4 in SI). The minimum and maximum of all survival predictions at each time point can be interpreted as a 95% uncertainty interval on the model predictions (reflecting parameter uncertainty), because the likelihood ratio test used α = 0.05. To compare the model predictions with experimental data involving a small number of individuals, we also simulated uncertainty originating from stochastic survival (i.e. the stochasticity of the death process itself). This was achieved by simulating the exact number of individuals in a given experimental treatment (e.g. 70 for malathion and 80 for carbendazim, cypermethrin and dimethoate in Figs 2 and 3) ten times with each combination of parameters (n = 10, see SI for details).

### Results

How well do the implementations GUTS-SD and GUTS -IT, calibrated on data from short-term toxicity experiments and constant exposure forecast effects of longer-term pulsed exposures? The likelihood values indicate a better fit to the calibration data for GUTS- SD (Table S14 in SI), but the majority of survival predictions are in better agreement with the data for GUTS-IT (Table S15 in SI). Accuracy and precision of the GUTS- IT and GUTS- SD model predictions of the effect pattern differed among chemicals and exposure regimes (Figs 2 and S1–S4 in SI). For malathion and carbendazim both models predicted too much mortality over time, whereas for cypermethrin, both models predicted too little mortality over time. For dimethoate, GUTS-SIC-IT predicted too little mortality over time, whereas GUTS-SIC-SD predictions agreed well with the data. When only considering effects at the end of the experiment (10 or 28 days), as is classically done for applications in environmental risk assessment, then the observed percentages of survivors are within the 95% confidence region of the model predictions in 8 out of 16 cases (Fig. 3 and panels D, F, J, L in Figs S1–S4 in SI).

### Discussion

Data points outside the confidence band occur frequently when fitting concentration-response curves to standard toxicity data. Thus data points outside the confidence-band of a forecasted concentration-response curve by GUTS, such as those in Fig. 3, may also be acceptable. Plotting of forecasted dose-response curves (Fig. 3) enables risk assessors to judge for themselves.

For GUTS-SD and GUTS-IT, standard ecotoxicity data is sufficient for model calibration. Using these models, we demonstrated that concentration-response curves can be generated for any desired exposure period, and that including time-varying exposure is possible (Figs 2 and 3). Furthermore, we could generate information about the time course of predicted survival (Fig. 2). This information has potential to complement the current information used for environmental risk assessment, which only focuses on concentration-response curves at the end of the experiment. In some of our simulations, the forecasted survival over longer durations than the calibration experiments tends to overestimate mortality (malathion, carbendazim, GUTS-SD prediction for dimethoate), whereas in others the forecasts underestimated mortality (cypermethrin, GUTS-IT prediction for dimethoate). This is in contrast with an earlier finding that GUTS- SD and GUTS-IT, calibrated on short-term tests tend to overestimate mortality in forecasts of longer exposure durations^16^. In another study using scaled damage as the dose metrics and 14 different chemicals, both GUTS -SD and GUTS -IT fit equally well to the data^20^. Overall, we suggest that given the current evidence, neither model is superior. In reality, survival likely follows a curve between these two limit cases of GUTS proper. With respect to application in environmental risk assessment, it cannot be known *a priori* which implementation will predict higher mortality or provide the best agreement with data under differing exposure patterns^21^. Indeed, the sensitivity of model output to changes in GUTS parameter values differs among exposure scenarios^21^, which partly explains why the IT and SD implementations provide different predictions under different scenarios. Hence it seems prudent to use both GUTS-SD and GUTS-IT (or GUTS proper, if sufficient data is available for calibration) for environmental risk assessment purposes. Furthermore, the worst case of GUTS-IT and GUTS-SD is in all of our cases either adequately predictive (i.e. the measured value at the end of the experiment is within the confidence region) or conservative (i.e. the worst case model overestimates mortality, Figs 2 and S1–S4 in SI).

A meta-analysis of existing, suitable datasets^16^,^20^,^31^,^32^ should be conducted to assess how well GUTS can forecast survival. Current guidance documents for environmental risk assessment of chemicals mention toxicokinetic-toxicodynamic models, such as GUTS, as a potentially useful method^24^,^33^. Educated forecasting requires mechanistic models, but more research is needed to establish how well the mechanisms in GUTS are able to provide sufficient predictive power. It is particularly worth investigating if toxicodynamic parameter values estimated from short term tests also apply to longer exposure durations. This is because toxicodynamic parameters reflect the toxic mode of action^20^, hence forecasting survival assumes the same toxic mode of action in short and longer exposures.

## Study 3: Interspecies variability of chemically-induced effects

**Figure 4:**
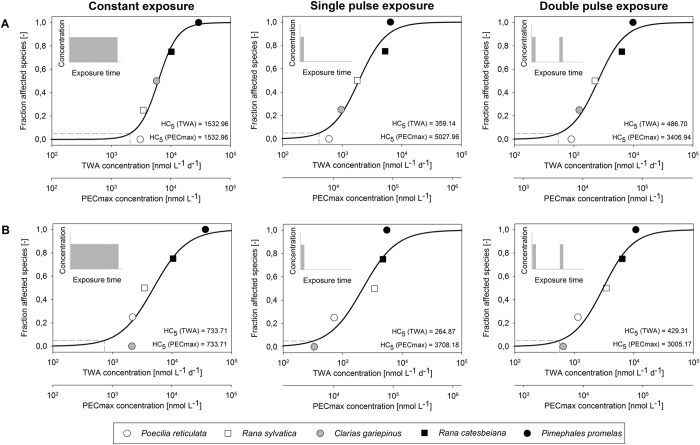
Species sensitivity distributions estimated for three exposure scenarios in computational experiments based on the toxicokinetic-toxicodynamic assumptions of scaled internal concentration: (**A**) stochastic death and (**B**) individual tolerance. The two x-axes correspond to the values of time weighted average (TWA) and maximum exposure (PECmax) concentrations for malathion.

Species sensitivity distributions (SSDs) are used to describe differences in sensitivity among species to a given chemical^34^,^35^,^36^,^37^. In case of mortality data, for each species, the concentration at which 50% of the tested individuals die under constant exposure and a given test duration (LC_50_ values), are plotted. This distribution represents the variability of species sensitivity within the studied taxonomic group, covering not only the species tested in the laboratory but also other species for which no data is available. The theoretical concentration affecting 5% of the species (HC_5_, Fig. 4) can be extracted from this distribution and is used in environmental risk assessment. Classical LC_50_ data (i.e. derived under constant exposure) may not be appropriate for environmental situations with time-varying exposure concentrations because they ignore the dynamics of exposure and effects and focus on cumulated effects at a single time point of the experiment (typically test termination)^38^,^39^. GUTS models allow estimating LC_50_ values that account for time-varying exposure and for how toxicity changes over time^3^,^39^,^40^. With that approach, time-varying exposure and effect patterns can be addressed more realistically. Since GUTS can predict any percent mortality (e.g. LC_10_), at any time point and for any given exposure profile, exposure-specific SSDs can be constructed when parameterized GUTS models are available for a sufficient number of species (e.g. a minimum of five to eight species is required in environmental risk assessment for vertebrates and invertebrates, respectively). We illustrate this new approach for building model-based SSDs using five vertebrate species exposed to malathion (Fig. 4).

### Methods

We collected survival data for five vertebrate species exposed to malathion, i.e. *Poecilia reticulata*^41^*, Rana sylvatica*^42^*, Clarius gariepinus*^43^*, Rana catesbeiana*^44^*, Pimephales promelas* 45, and used it for model calibration. We calibrated GUTS -SD and GUTS -IT models with scaled internal concentration as the dose metric (see Tables S16 and S17, Figs S5 and S6 in SI). Then we used the models to calculate 14-day LC_50_s for constant exposure and for two different pulse exposure scenarios (single and double pulses). These LC_50_ values were then used as input data for the SSD calculations. In order to construct the SSD, effects in a given species (as represented by its LC_50_ value) need to be statistically related to exposure (i.e. concentration in water), for which different surrogates may be used. In this study, LC_50_ values were related to exposure using either the maximum predicted exposure concentration (PEC_max_) or the time weighted average (TWA)^24^. The SSD was obtained by statistical fitting to the LC_50_ data for a given exposure surrogate, and the corresponding HC_5_ (5% percentile of the distribution) was estimated. This work was repeated for (i) LC_50_ values generated either with GUTS-IT or GUTS-SD, (ii) the two selected exposure surrogates and (iii) various exposure patterns: constant or pulsed exposure (Fig. 4).

### Results

We evaluated the impact of the chosen GUTS model, concentration surrogate and exposure pattern on the SSDs and corresponding HC_5_ values. The HC_5_ estimates differed among exposure scenarios, among exposure concentration surrogates (TWA and PEC_max_) and between GUTS- IT and GUTS- SD (Fig. 4). For a given GUTS model, the HC5 estimates at constant exposure are identical when calculated based on TWA and PEC_max_. With pulsed exposure patterns, the HC_5_ calculated based on the TWA increases with the number of pulses, whereas it decreases when PEC_max_ is used. In this example for malathion and five vertebrate species, the same time weighted average concentration but different temporal profiles lead to different SSDs and thus HC_5_ estimates; the constant exposure is less toxic than the shorter but higher equivalent pulse exposure. The GUTS-SD model predicts that two species swap ranks in the SSD depending on the exposure type. For constant exposure *Rana sylvatica* is predicted to be more sensitive than *Clarias gariepinus*, whereas for pulsed exposures it is the other way round (Fig. 4A). Also the SSDs differ between GUTS-SD, where *Poecilia reticulata* is the most sensitive species (Fig. 4A), and GUTS-IT (Fig. 4B) where *Clarias gariepinus* is the most sensitive species.

### Discussion

All these results arise from the interplay of exposure, toxicokinetics and toxicodynamics and cannot easily be generalized to other combinations of species and chemicals. A mechanistic interpretation of the GUTS model parameters (Tables S16 and S17) can help to explain our observations, but requires further, systematic investigations before we can draw general conclusions. We present a method combining mechanistic modelling with a statistical approach (SSD fitting) for assessing the changes in SSDs in response to changes in the exposure scenario, more specifically for time-varying stressor intensities. In this example, the GUTS- SD implementation resulted in higher HC_5_ estimates than those derived from the GUTS -IT, but this cannot be generalized. Either GUTS -IT or GUTS -SD can result in stronger effects compared to its respective counterpart^21^, because the outcomes (SSD and HC_5_ values) depend on the intrinsic properties of the two toxicodynamic assumptions (IT or SD)^20^ and the characteristics of the exposure profile^21^. We do not know how the mechanisms of toxicity and the choice of species influence survival predictions across different exposure patterns, but suspect all three aspects (chemical, species, exposure pattern) are intricately linked.

Our modelling suggests that differences in species responses for various exposure scenarios can lead to differences in the ranking of the species in the SSD and also in the estimated HC_5_ values (Fig. 4) when the exposure concentration is not constant. Thus, when toxicity is assessed for time-varying exposure concentrations, the data analysis needs to account for the possibility that species may differ in their responses to different exposure patterns. Further studies should investigate whether such a dependence of SSDs on exposure regimes can also be found for other species and chemicals and seek experimental confirmation of our modelling results. This study suggests that the temporal pattern of exposure needs to be considered when interpreting the results of an SSD where exposure is not constant. Importantly, the SSD and associated HC_5_ estimate could differ for exposures with the same time weighted average concentration but different temporal profiles (i.e. reciprocity or Haber’s law do not apply).

The proposed method for building SSDs allows assessing risks of untested exposure patterns based on standard datasets for a range of species. We suggest that the approach could be used in future environmental risk assessments of chemicals to derive species sensitivity distributions for more realistic exposure patterns, such as FOCUS surface water scenarios^46^ or measured concentration time series^47^. The GUTS model framework offers the possibility to derive reliable effect measures such as the HC_5_ for untested exposure scenarios, to quantify uncertainties, and has the potential for cross species extrapolation.

We did not attempt to explain the species sensitivity differences by taking advantage of the biological interpretation of GUTS parameters. Some studies hypothesized that TKTD model parameters, such as those of GUTS models, could be combined with species traits or phylogenetic information to explain and predict species sensitivity differences^48^,^49^. Species traits such as metabolic rate, which scales with size^50^, correlated with the threshold parameter for a small set of chemicals^51^ and the dominant rate constant could be related to the size of three different species^52^. These two examples suggest that predictions of species sensitivity based on phylogeny^49^,^53^,^54^ could be refined to predict GUTS parameters as proxy for sensitivity. Such new statistical models should then be tested on a wide range of species and chemicals. The hope is that predictions can be made for untested species based on correlations between species traits and TKTD parameters^48^,^55^. Building these predictive models requires that the parameters involved have a biological meaning and that the parameters can be linked to quantifiable traits/quantities. For this purpose, the GUTS models based on scaled internal concentration might be inappropriate, as scaled internal concentration cannot be directly measured and lumps very different processes, e.g. chemical elimination and damage repair^3^. Both elimination and damage recovery processes may have a strong impact on an organism’s survival to a degree depending on the properties of the chemical and species tested. However, they are likely linked to very different species traits. Therefore it is important to differentiate between toxicokinetics and toxicodynamics^20^ when building inter-species toxicity extrapolation models^53^,^56^, for example by using internal concentrations as a driving variable and scaled damage as a dose metric (Table 1). This approach may provide better opportunities to develop a trait-based predictive framework for ecotoxicity^48^.

## Conclusions

GUTS models have already been applied to predict survival under time-varying exposure^16^,^21^, model starvation resistance^57^, model combined effects of toxicity and starvation^58^, represent temporal variation in toxicity in an individual-based model^22^,^59^, link temporal biomarker response to survival^60^, model survival of gill cells *in vitro*^61^, map toxicodynamic parameters in chemical space^20^,^62^, approximate toxicodynamic recovery times^20^ and investigate sensitivity differences between species^52^,^63^,^64^ and life-stages^52^,^65^,^66^.

The commonly asked question in survival analysis is how a stressor affects survival over time and how we could predict it. We found that forecasting survival across different exposure patterns does not necessarily require internal concentrations. Instead, measured or modelled external concentrations can be used as driving variables in conjunction with scaled internal concentrations as a dose metric^16^,^21^. GUTS survival forecasts can help bridge the gap between laboratory and field situations and make environmental risk assessment of chemicals more relevant and realistic^67^,^68^,^69^. Importantly our results show the interdependence of species sensitivity and exposure patterns, which calls not only for a systematic investigation but also a stronger integration of exposure and effect assessments.

Some questions, for example why species differ in their sensitivity to chemicals, may require a more detailed approach than using scaled internal concentrations as a dose metric. Here, the GUTS framework offers a consistent set of models and equations for separate quantification of toxicokinetics and toxicodynamics. To achieve that, measured or modelled internal concentrations can be used in conjunction with scaled internal damage^20^. The resulting toxicodynamic parameter values can be linked to chemical class^20^,^62^, but also toxicodynamic modelling is the organism level equivalent of quantitative adverse outcome pathways^70^. Here, the GUTS framework offers a starting point to develop similarly coherent toxicokinetic-toxicodynamic models for sub-lethal endpoints.

The GUTS framework enables us to analyze situations where the stressor intensity changes over time and this can also be applied to questions beyond toxicology, for example in medicine or engineering. GUTS models also allow the quantification of the deterministic and stochastic component of death or failure or occurrence of an event. We expect that a wide range of scientific questions could benefit from applying GUTS and hope that our analysis, from an ecotoxicology perspective, can serve as a blueprint for other fields of science. To facilitate further studies we provide extensive model code and software (http://www.ecotoxmodels.org/guts/).

## Additional Information

**How to cite this article**: Ashauer, R. *et al*. Modelling survival: exposure pattern, species sensitivity and uncertainty. *Sci. Rep.* **6**, 29178; doi: 10.1038/srep29178 (2016).

## Supplementary Material

Supplementary Information

## Figures and Tables

**Table 1:** GUTS flavors.

Abbreviation	Driving variable	Dose metric	Rate constant & interpretation	Typically used when
GUTS-SIC-SD, GUTS-SIC-IT	External concentration	Scaled Internal Concentration	*k*_e_, TK&TD recovery (“dominant rate constant”)	Internal concentrations not available
GUTS-SID-SD, GUTS-SID-IT	Internal concentration	Scaled Internal Damage	*k*_r_, TD recovery (“damage recovery rate constant”)	Measured or modelled internal concentrations available
GUTS proper-SIC	External concentration	Scaled Internal Concentration	*k*_d_, TK&TD recovery (“dominant rate constant”)	Internal concentrations not available
GUTS proper-SID	Internal concentration	Scaled Internal Damage	*k*_r_, TD recovery (“damage recovery rate constant”)	Measured or modelled internal concentrations available

The most suitable model flavor depends on the question at hand and data available. A detailed derivation and discussion can be found in previous publications^3^,^16^,^20^.
