# Supplementary material for: Modelling survival: exposure pattern, species sensitivity and uncertainty
Source: Sci Rep. 2016 Jul 6;6:29178. doi: 10.1038/srep29178 (PMC4933929; doi:10.1038/srep29178)
Supplement: Supplementary Information [file srep29178-s1.pdf]

Supporting Information to

Modelling survival: exposure pattern, species sensitivity and uncertainty

Roman Ashauer*, Carlo Albert, Starrlight Augustine, Nina Cedergreen, Sandrine Charles, Virginie Ducrot, Andreas Focks, Faten Gabsi, André Gergs, Benoit Goussen, Tjalling Jager, Nynke I. Kramer, Anna-Maija Nyman, Veronique Poulsen, Stefan Reichenberger, Ralf B. Schäfer, Paul J. van den Brink, Karin Veltman, Sören Vogel, Elke I. Zimmer, Thomas G. Preuss

*Corresponding author, Email: roman.ashauer@york.ac.uk, Phone: +44-(0)-1904-324052

www.ecotoxmodels.org/GUTS

Contents

Example code and software 3

Mathematica 3

Matlab 3

GUTS R package 3

GUTS implementation with graphical user interface 3

Implementation in ModelMaker 4

GUTS-SIC-SD and GUTS-SIC-IT 4

Time course of dose metric 4

Link to survival in GUTS-SIC-SD 4

Link to survival in GUTS-SIC-IT 5

Parameter optimisation used for forecasting survival across different exposure patterns 6

Calculation of model performance statistics 6

Numerical estimation of parameter confidence intervals 7

Stochasticity of survival 7

Including parametric uncertainty in forecasts 8

Probabilistic modelling of survival over time 9

Experiments with *G. pulex* 10

Calibration data of *G. pulex* survival 10

G. pulex survival data under pulsed exposure 11

Construction of simulated concentration-response relationships 15

Concentration-response curves for constant exposures 15

Concentration-response curves for pulsed exposures ... 15
Best fit parameters ... 16
Performance statistics for model predictions ... 17
Additional figures of survival predictions across different exposure patterns ... 18
Detailed assessment of survival predictions across exposure patterns ... 22
Background ... 22
Calibration ... 22
Prediction ... 22
Materials and methods used for the calculation of interspecies variability of effects ... 24
Data and model calibration ... 24
Best fit parameters for the different species ... 24
Survival plots and scaled internal concentrations for the different species ... 25
Calculations on synthetic data with Matlab ... 28
What is a 'slow' dominant rate constant? ... 30
References ... 34

Example code and software

All example code and software can be downloaded free of charge as supporting information to this publication.

Mathematica

Model calibration, predictions and estimation of uncertainty bands to forecast survival of *G. pulex* across different exposure patterns were performed in Mathematica 10.0 (Wolfram Research). This is an example implementation of GUTS-SIC-IT and GUTS-SIC-SD, where the IT version assumes a log-logistic distribution of the threshold. The Mathematica code is provided as separate supporting information.

Matlab

Additional calculations on the synthetic data (see sections below) were made with the BYOM package for Matlab and the GUTS2 package (which can be used for all GUTS flavours). Both can be downloaded free of charge from http://www.debtox.info/byom.html. The BYOM package applies a Simplex optimisation routine (which can be easily changed to other Matlab-based optimisation functions) for the likelihood function (following from the multinomial distribution[1]). To produce confidence intervals, the BYOM package includes likelihood profiling[2] and MCMC sampling (yielding a Bayesian posterior that is used to derive marginal distributions for individual parameters, as well as the joint distribution for forward prediction). Example fits are shown in the sections below.

GUTS R package

Inference with GUTS proper on the synthetic data was performed with the R-package "GUTS" [3,4]. A log-normal distribution of thresholds was used [i]. The priors were chosen to be flat. The posterior samples were generated by means of adaptive Monte Carlo chains (R package "adaptMCMC"). To get a start value for the chains, the optimizer "hjkb" from the R-package "dfoptim" was employed. Since the posterior marginals of some of the parameters seem to have non-vanishing support at infinity, all parameters were transformed with exp(-ϑ) before running the chains. Chains of length 1 M (resp 2 M) were used and the adaptation phase (10 % of the chain) was discarded. The maximum of the posterior (best fit) as well as 2.5% and 97.5 % quantiles of the posterior marginals are plotted in Figure 3.

The R-package can be downloaded free of charge from http://cran.r-project.org/web/packages/GUTS/index.html. To demonstrate the fits on the synthetic data sets, two examples are shown in Figure S9.

GUTS implementation with graphical user interface

An example implementation of GUTS-SIC-IT and GUTS-SIC-SD in Delphi (http://www.embarcadero.com/products/delphi) is also provided. For the IT version a log-logistic, log-normal or Weibull distribution of the threshold can be selected, although the log-logistic distribution is recommended. The optimal parameter set is found by optimizing the likelihood function for GUTS (see Eq. 8) and parameter confidence intervals are calculated using likelihood

[i] We do not see any strong reasons to prefer one threshold distribution over the other (e.g. log-normal vs log-logistic), however the log-logistic distribution is easier to implement computationally in some software environments. In this study we used the log-logistic distribution in the Mathematica and ModelMaker implementations and log-normal in R and Matlab.

profiling. The best optimization results are usually obtained with Simulated Annealing, but the fastest optimization is typically achieved using Downhill Simplex. The code for both these algorithms originates from the TPmath library (Version 0.81, 2011 http://sourceforge.net/projects/tpmath/). The implementation comes with a graphical user interface, needs no installation and has been used previously[5-8].

Implementation in ModelMaker

An example implementation of GUTS-SIC-IT and GUTS-SIC-SD in ModelMaker is also provided and can be downloaded free of charge from http://www.ecotoxmodels.org/guts/. The IT version assumes a log-logistic distribution of the threshold. ModelMaker (http://www.modelkinetix.com/) is a user friendly software that is well suited to model prototyping, differential equations and model calibration. The ModelMaker implementation includes the likelihood function for GUTS (Eq. 8) and uses the Downhill Simplex algorithm for parameter estimation. GUTS-SIC-IT, GUTS-SIC-SD, GUTS-SID-IT and GUTS-SID-SD versions have been used previously[9-11].

GUTS-SIC-SD and GUTS-SIC-IT

All the models we used were previously published[1,10]. We recall them here for clarity.

Time course of dose metric

We use the scaled internal concentration $C_i^*(t)$ as a dose metric, which is the true internal concentration divided by the bioconcentration factor[1]. This TK model can be used in absence of information on body residues; it should be noted that C_i^* has the dimension of an external concentration. The time course of the scaled internal concentration is determined by the dominant rate constant k_e (time^{-1}) and given by the differential equation:

$$\frac{dC_i^*(t)}{dt} = k_e\left(C_w(t) - C_i^*(t)\right) \qquad \text{Eq. 1}$$

The driving variable or model input is the external concentration in water $C_w(t)$. The scaled internal concentration $C_i^*(t)$ is linked to the stochastic death (SD) or individual tolerance (IT) model to describe the survival over time[1,10].

Link to survival in GUTS-SIC-SD

In the SD model, the hazard rate, or the instantaneous probability to die, is calculated from the scaled internal concentration:

$$h_z(t) = k_k \max\left(0, C_i^*(t) - z\right) + h_b \qquad \text{Eq. 2}$$

which describes hazard increasing in proportion to how much the dose metric exceeds the threshold z (concentration). The killing rate constant k_k (concentration^{-1}.time^{-1}) is the proportionality constant and h_b (time^{-1}) the background hazard rate.

For the parameterisation of the SD model, the killing rate constant and the internal threshold have to be estimated from the survival data. As the dose metric is the scaled internal concentration, z and k_k will have the dimension of external concentrations.

The survival probability $S_{SD}(t)$ is the probability of individual to survive until time t. It is calculated as follows:

$$S_{SD}(t) = e^{-H_z(t)} \text{ with } H_z(t) = \int_0^t h_z(\tau)\,d\tau \qquad \text{Eq. 3}$$

The background hazard rate should be fitted on the entire dataset, although sometimes it can be defensible to fit it only on the control survival data.

Link to survival in GUTS-SIC-IT

In the IT model we assume that the threshold follows a probability distribution, such as the log-logistic (or log-normal or other suitable distribution)[ii], and that death is immediate when the scaled internal concentration exceeds the individual's threshold. We can calculate the survival probability for an individual following a cumulative log-logistic distribution of the threshold z by[10]:

$$F(t) = \frac{1}{1+\left(\max_{0\le\tau\le t} C_i^*(\tau)/\alpha\right)^{-\beta}} \qquad \text{Eq. 4}$$

where α is the median of the distribution of z (concentration), and β (-) is the shape parameter of the distribution and $F(t)$ is the cumulative log-logistic distribution of the threshold z.

Instead of the shape parameter, an easy-to-interpret alternative can be used in the form of a 'factor of spread' F_s. This is the factor that needs to be used on the median value of z to cover 95% of the probability distribution (i.e., from the 0.025-0.975 percentile). This parameter can be used for all distributions that are symmetrical on log-scale (also for the log-normal distribution). For the log-logistic distribution, β equals $\ln(39)/\ln(F_s)$, and for the log-normal distribution, the standard deviation on $\log_{10}$ scale equals $\log_{10}(F_s)/1.96$.

In the IT model, the survival is related to the maximum dose metric that individuals experienced until time t rather than to the actual value of the dose metric at time *t*, because organisms that died previously remain dead (this is particularly important when dealing with time-varying exposure). The probability to survive until time t is then:

$$S_{IT}(t) = \left(1 - F(t)\right)e^{-h_b t} \qquad \text{Eq. 5}$$

[ii] For the synthetic data to optimize experimental design for calibration of the GUTS proper, a log-normal distribution was used.

Parameter optimisation used for forecasting survival across different exposure patterns

Survival probabilities for both the SD and the IT model are calculated in explicit dependence of the parameter vector θ and the external concentration over time, so formally

$$S_{SD}(t) = S_{SD}(\theta, C_w, t) = S_{SD}(\{k_k, z, k_e, h_b\}, C_w(t), t) \qquad \text{Eq. 6}$$

and

$$S_{IT}(t) = S_{IT}(\theta, C_w, t) = S_{IT}(\{\alpha, \beta, k_e, h_b\}, C_w(t), t) \qquad \text{Eq. 7}$$

Survival data follow a multinomial distribution, hence the following log-likelihood function applies[1]:

$$\ln l(\theta|\mathrm{y}) = \sum_{j=1}^{n+1} (y_{j-1} - y_j) \ln(S_{j-1}(\theta) - S_j(\theta)) \qquad \text{Eq. 8}$$

Where y_j are experimental observations at sampling times j and S_j are simulated survival probabilities at sampling time j given parameter vector θ. The maximum of the log-likelihood function indicates an optimal fit between observed and simulated survival. In practice, the negative of the log-likelihood function is often used because parameter estimation algorithms usually minimise the objective function. Here we used:

Objective function = $\ln l(\theta|y)$. Eq. 9

The smaller the objective function, the better is the fit.

To find the optimal vector of parameter values θ_{opt} for each of the SD and the IT models, we optimised the parameter values with respect to the experimentally observed survival data. We used the built-in optimisation routine *Simulated Annealing* of the method *NMinimize* of Mathematica (Wolfram Research, version 10.0) to obtain the best fit between data and model simulations by minimizing $-\ln l(\theta|y)$, i.e. maximizing the log-likelihood function itself.

The optimisation routine yielded an optimal parameter vector θ_{opt} for which the log-likelihood function shows the highest values $\ln l(\theta|y)$. Optimisation was repeated twice after the first optimisation run, each time using the optimum values from the previous optimisation step as starting values, finally resulting in an optimal parameter set θ_{opt} for each species and model version. All parameter vectors θ being tested during the optimisation routine have been stored together with the resulting likelihood values $\ln l(\theta|y)$ for further analyses.

Calculation of model performance statistics

For the quantification of the accuracy of the model predictions, we used the chi-square as measure of model performance

$$\chi^2 = \sum_{j=1}^{n} \frac{(y_j - \hat{y}_j)^2}{\hat{y}_j + 1} \qquad \text{Eq. 10}$$

where y_j indicate observed numbers of surviving individuals and $\hat{y}_j$ the predicted number of surviving individuals (not to be confused with the survival rates) at sampling times j and n the number of observation points. Addition of 1 in the denominator is corresponding to division of surviving individuals into classes: class n for numbers of surviving individuals from $n-1$ to n. Since the test statistic only asymptotically follows a χ^2 distribution, and the expected counts in some bins will be low, the result should be considered an approximate measure for goodness-of-fit.

Numerical estimation of parameter confidence intervals

The likelihood ratio method was used to estimate confidence intervals for the optimal parameters[2,12] θ_{opt}. Confidence intervals for the single parameters for a given confidence level α were calculated as the profile likelihood. The confidence set includes those parameter values for which the likelihood ratio fulfils the condition:

$$2\left[\ln l(\theta|y) - \ln l(\theta_{(p)}|y)\right] \leq \chi^2_{df,1-\alpha} \qquad \text{Eq. 11}$$

with $\chi^2_{df,1-\alpha}$ being the value of the Chi-square distribution for the confidence level α, and the degrees of freedom of the likelihood ratio. For single parameter confidence intervals, the likelihood ratio has $df = 1$. To find these parameter values, one value in the parameter vector, say the pth parameter θ_p was set to successively decreasing values (the vector $\theta_{(p)}$ then denotes all parameters except *p*), starting at the maximum-likelihood estimate, i.e. the best parameter value. All parameter values with exception of parameter θ_p were again optimised (using the *NMinimize* method and the *SimulatedAnnealing* algorithm in Mathematica) to give a best fit to the experimental data. This procedure was repeated until the value of $\ln l(\theta|y)$ satisfied eq. 11. Likewise, this procedure was repeated for successively increasing parameter values, again starting with the optimal maximum-likelihood estimate. The first parameter values that did not fulfil condition (eq. 11) were taken as lower and upper confidence interval limits to the confidence level α. This procedure was performed for all parameters and $\alpha = 0.05$. All parameter vectors $\theta_{(p)}$ being tested during the confidence interval estimation routine have been stored together with the resulting likelihood values for further analyses.

Stochasticity of survival

Survival is a binary process, i.e. an individual can either be alive or death. The number of individuals in a group of individuals influences the uncertainty of model predictions about the survival of the group of individuals, especially for small numbers ($n < 100$). To consider the specific nature of survival, survival is modelled as a binomial process. Multinomial distributions have previously been used to model survival[1] and they are equivalent to the conditional binomial distribution, but only when there are no animals lost to follow-up[13]. Here we model survival as a binomial process, with the number of surviving individuals being proportional to the conditional binomial distribution:

$$y_{t+\Delta t} \sim B\left(\pi_{t+\Delta t}, y_t\right) \qquad \text{Eq. 12}$$

where $y_{t+\Delta t}$ is the number of survivors in a population at time $t+\Delta t$ and y_t the number of individuals being alive at time t. $\pi_{t+\Delta t}$ is the conditional probability to survive from time t until time $t+\Delta t$ given by:

$$\pi_{t+\Delta t} = \frac{S\left(\theta, C_w, t+\Delta t\right)}{S\left(\theta, C_w, t\right)} \qquad \text{Eq. 13}$$

where $S\left(\theta, C_w, t\right)$ is the survival probability calculated by the SD or the IT model for parameter vector θ, external concentration time course C_w, and the time t. $S\left(\theta, C_w, t\right)$ is the probability to survive until time t, which is different to the conditional probability of Eq. 13. Note that the time step is not fixed to 1 here, as the survival probabilities can be evaluated for arbitrary small or large time steps, hence conditional probabilities can be modelled as a random process using a flexible time step Δt.

For a parameter vector θ, a chosen external concentration regime C_w and an initial population size y_0, the number of surviving individuals in the population is modelled in an iterative way, starting with y_0 individuals at $t=0$, by drawing for every new time step $t+\Delta t$ from a binomial distribution parameterised by the number of living organisms at time t, and the calculated survival rates for times t and $t+\Delta t$ as given in Eq. 12 and 13. By performing $n_{S\max}$ repetitions of this procedure, we obtain $n_{S\max}$ realisations of the survival probabilities within a population of initial size y_0, given parameter vector θ and external exposure C_w, from which statistical descriptors such as median and percentiles are calculated.

Including parametric uncertainty in forecasts

Parametric uncertainty in model simulations is in addition to the stochasticity of survival itself. To consider parametric uncertainties, the following approach is used.

For all monitored parameter vectors $\theta_{(p)}$ that have been generated and tested within the parameter optimisation and confidence interval estimation routines, the corresponding likelihood values $\ln l(\theta_{(p)}|y)$ have been stored. To approximate the joint confidence regions for the parameters, we selected those parameter sets from the optimisation procedure (simulated annealing) that were not rejected in a likelihood ratio test (critical value 7.81 from Chi-square distribution, $df=3$, $\alpha=0.05$):

$$2\left[\ln l(\theta|y) - \ln l(\theta_{(p)}|y)\right] \le \chi^2_{df,1-\alpha} \qquad \text{Eq. 14}$$

with $\chi^2_{df,1-\alpha}$ being the value of the Chi-square distribution for the confidence level α, and the degrees of freedom of the likelihood ratio. The degrees of freedom for the likelihood ratio that is being used for the construction of the joint confidence region are defined by the difference in free model parameters, that is in this case $df=3$, because for optimisation all three model parameters of the GUTS-SIC-SD or GUTS-SIC-IT models were free, whereas for the construction of the three-dimensional joint confidence region all model parameters were fixed. The background mortality rate constant was not taken into account here, because it was not considered for the uncertainty calculations as purpose of the forecasts is to inform about the effect of chemical stress on survival.

Probabilistic modelling of survival over time

Information about parameter uncertainty and about stochasticity of survival were combined to characterise uncertainties of model simulations. For a given exposure C_w, a number of $n_{U\max}$ parameters from the confidence region θ_{conf} were drawn, and for every parameter vector $\theta_{(p)}$ the survival over time was simulated as an explicit conditional binomial process in $n_{S\max}$ iterations. The total numbers of simulations hence amounts to $n_{\max} = n_{U\max} n_{S\max}$. These $n_{\max}$ realisations of the simulated numbers of surviving individuals within the population of initial size y_0 can be described statistically, e.g. by the median and minimum and maximum of the distribution of the numbers of survivors, quantifying uncertainty in the model predictions. For this study, we used $n_{S\max} = 10$, $n_{U\max} = 1000$ for malathion and carbendazim, and $n_{U\max} = 500$ for cypermethrin and dimethoate; hence we performed $n_{\max} = 10000$ and $n_{\max} = 5000$ simulations of survival over time, respectively.

Experiments with *G. pulex*

The toxicity tests with *G. pulex* and carbendazim, dimethoate and cypermethrin followed previously established methods and protocols[10,11,14,15]. Adult test organisms were collected in the field (Itziker Ried, Greifensee catchment, near Zurich, Switzerland), acclimatised to laboratory conditions (e.g. 13°C) and fed with pre-conditioned horse-chestnut leaves. Toxicity experiments were conducted in 600 mL pyrex beakers filled with 500 mL of pre-aerated artificial pond water. At the start of the experiments each beaker contained 10 individual *G. pulex*, which were fed with pre-conditioned horse-chestnut leaf discs *ad libitum*. Survival was observed daily. Conductivity, pH and dissolved oxygen content were monitored.

We used 14-C labelled test chemicals to enable measurement of exposure concentrations via liquid scintillation counting. The short experiments (4 day duration) consisted of seven treatments and controls, where each treatment consisted of two replicate beakers (i.e. 20 individuals initially per treatment). The treatments in the longer experiments consisted of pulsed exposure with short interval (two pulses, 8 replicate beakers), pulsed exposure with longer interval (two pulses, 8 replicate beakers), constant exposure (8 replicate beakers), solvent controls (mimicking long interval pulsed exposure pattern, 4 beakers) and controls (mimicking long interval pulsed exposure pattern, 4 beakers). At the end of pulses and at regular water renewables, organisms were rinsed and then placed into new beakers with fresh artificial pond water.

Calibration data of *G. pulex* survival

The acute toxicity data for Malathion were previously published elsewhere[15]. We repeat them here for completion.

Table S1: Number of alive *G. pulex* in each treatment during short toxicity test with malathion.

Days	3.837 nmol/L	2.805 nmol/L	1.867 nmol/L	1.148 nmol/L	0.739 nmol/L	0.543 nmol/L	0.345 nmol/L	controls
0	20	20	20	20	20	20	20	20
1	20	20	20	20	20	20	20	20
2	9	12	19	19	20	20	20	19
3	1	5	10	12	19	20	20	19
4	0	2	3	6	17	20	20	19

Table S2: Number of alive *G. pulex* in each treatment during short toxicity test with carbendazim.

Days	1279.8 nmol/L	447.9 nmol/L	156.8 nmol/L	54.9 nmol/L	19.2 nmol/L	6.7 nmol/L	2.4 nmol/L	controls
0	20	20	20	20	20	20	20	20
1	17	18	16	16	19	16	19	19
2	10	11	15	15	16	14	18	17
3	4	8	11	14	12	13	17	17
4	0	5	8	14	11	13	16	16

Table S3: Number of alive *G. pulex* in each treatment during short toxicity test with cypermethrin.

Days	1.9224 nmol/L	0.7113 nmol/L	0.2632 nmol/L	0.0974 nmol/L	0.036 nmol/L	0.0133 nmol/L	0.0049 nmol/L	controls
0	20	20	20	20	20	20	20	19
1	0	1	6	20	20	20	20	18
2	0	0	0	9	19	19	19	18
3	0	0	0	6	19	19	19	18
4	0	0	0	2	15	19	18	17

Table S4: Number of alive *G. pulex* in each treatment during short toxicity test with dimethoate.

Days	218529 nmol/L	87412 nmol/L	34965 nmol/L	13986 nmol/L	5594 nmol/L	2238 nmol/L	895 nmol/L	controls
0	20	20	20	20	20	20	20	20
1	11	18	18	20	20	19	19	19
2	1	3	7	18	20	18	18	19
3	0	1	3	15	17	18	18	19
4	0	0	1	9	16	16	16	19

G. pulex survival data under pulsed exposure

The pulsed toxicity data for malathion were previously published elsewhere[11]. We show the data from treatments A and B here, but note that the original data also contain an additional treatment C which was not used in this study.

Table S5: Concentration of malathion in the treatments during the pulsed exposure experiment. The original data[11] also contain an additional treatment C which was not used here.

Treatment A (pulsed, short interval)	Treatment A (pulsed, short interval)	Treatment B (pulsed, long interval)	Treatment B (pulsed, long interval)
Time (days)	Concentration (nmol/L)	Time (days)	Concentration (nmol/L)
0.0000	4.0262	0.0000	4.0243
0.0071	4.0262	0.0071	4.0243
0.9929	3.7924	0.9929	3.7898
1.0029	0.0000	1.0029	0.0000
6.1458	0.1280	7.1701	0.1597
6.1632	3.9464	9.0035	0.0000
7.1701	3.8247	9.0104	4.0264
7.1801	0.0000	10.0035	3.8358
10.0035	0.1045	10.0135	0.0000
15.0174	0.0000	15.0174	0.1044
17.0104	0.0000	17.0104	0.0000
27.0174	0.0000	27.0174	0.0000
27.1000	0.0000	27.1000	0.0000

Table S6: Number of alive *G. pulex* in each treatment during long toxicity test with malathion. The original data[11] also contain an additional treatment C which was not used here.

Days	Treatment A (pulsed, short interval)	Treatment B (pulsed, long interval)
0	70	70
1	67	69
2	63	66
3	62	66
4	61	65
5	60	65
6	60	64
7	54	62
8	45	61
9	43	61
10	40	48
11	37	43
12	36	39
13	33	37
14	32	33
15	30	32
16	29	31
17	25	27
18	23	26
19	23	25
20	21	25
21	20	24
22	20	24

Table S7: Concentration of carbendazim in the treatments during the pulsed exposure experiment. Note that treatment C was not used in the figures due to space limitations.

Treatment A (pulsed, short interval)	Treatment A (pulsed, short interval)	Treatment B (pulsed, long interval)	Treatment B (pulsed, long interval)	Treatment C (constant exposure)	Treatment C (constant exposure)
Time (days)	Concentration (nmol/L)	Time (days)	Concentration (nmol/L)	Time (days)	Concentration (nmol/L)
0	0	0	0	0	0
0.003	6914.14	0.005	7071.00	0.005	1189.96
1.000	6802.85	1.000	6919.75	3.014	1198.96
1.01	0	1.01	0	3.028	1166.26
2.000	0.00	3.031	0.00	7.035	1144.84
2.010	6933.66	6.042	0.00	7.038	1124.64
3.000	6798.91	6.049	6920.60	10.100	1124.64
3.010	0.00	7.042	6861.19		
6.035	0.00	7.052	0.00		
10.014	0.00	10.031	0.00		
10.100	0.00	10.100	0.00		

Table S8: Number of alive *G. pulex* in each treatment during long toxicity test with carbendazim. Note that treatment C was not used in the figures due to space limitations.

Days	Treatment A (pulsed, short interval)	Treatment B (pulsed, long interval)	Treatment C (constant exposure)	Controls
0	80	80	80	80
1	78	79	78	77
2	59	40	71	74
3	31	28	40	74
6	7	24	5	67
7	7	21	2	65
8	6	11	0	65

Table S9: Concentration of cypermethrin in the treatments during the pulsed exposure experiment. Note that treatment C was not used in the figures due to space limitations.

Treatment A (pulsed, short interval)	Treatment A (pulsed, short interval)	Treatment B (pulsed, long interval)	Treatment B (pulsed, long interval)	Treatment C (constant exposure)	Treatment C (constant exposure)
Time (days)	Concentration (nmol/L)	Time (days)	Concentration (nmol/L)	Time (days)	Concentration (nmol/L)
0	0	0	0	0	0
0.010	0.100	0.010	0.107	0.010	0.017
1.017	0.050	1.031	0.044	3.031	0.009
1.027	0.000	1.041	0.000	3.045	0.010
2.042	0.004	3.017	0.002	7.063	0.000
2.049	0.116	6.135	0.000	7.069	0.011
3.049	0.063	6.142	0.114	10.115	0.008
3.059	0.000	7.149	0.070		
6.115	0.000	7.159	0.000		
10.115	0.000	10.115	0.000		

Table S10: Number of alive *G. pulex* in each treatment during long toxicity test with cypermethrin. Note that treatment C was not used in the figures due to space limitations.

Days	Treatment A (pulsed, short interval)	Treatment B (pulsed, long interval)	Treatment C (constant exposure)	Controls
0	80	80	80	80
1	76	65	78	79
2	74	62	76	79
3	19	61	75	77
6	14	49	72	73
7	14	40	71	73
8	13	37	68	72
9	13	34	65	72
10	13	33	64	71

Table S11: Concentration of dimethoate in the treatments during the pulsed exposure experiment. Note that treatment C was not used in the figures due to space limitations.

Treatment A (pulsed, short interval)	Treatment A (pulsed, short interval)	Treatment B (pulsed, long interval)	Treatment B (pulsed, long interval)	Treatment C (constant exposure)	Treatment C (constant exposure)
Time (days)	Concentration (nmol/L)	Time (days)	Concentration (nmol/L)	Time (days)	Concentration (nmol/L)
0	0	0	0	0	0
0.010	44931	0.010	44704	0.010	9141
1.017	43830	1.028	44007	3.038	9180
1.027	0	1.038	0	3.042	9387
1.997	121	3.028	0	7.014	9340
2.000	41998	5.997	0	7.017	8191
3.000	44433	6.003	41332	10.028	9255
3.010	0	7.000	44835		
6.035	0	7.010	0		
10.028	0	10.028	0		

Table S12: Number of alive *G. pulex* in each treatment during long toxicity test with dimethoate. Note that treatment C was not used in the figures due to space limitations.

Days	Treatment A (pulsed, short interval)	Treatment B (pulsed, long interval)	Treatment C (constant exposure)	Controls
0	80	80	80	80
1	71	56	78	79
2	51	38	61	78
3	42	31	34	76
6	30	20	24	71
7	27	15	19	67
8	21	9	18	67
9	16	6	14	67
10	10	3	12	67

Construction of simulated concentration-response relationships

Concentration-response curves for constant exposures

Simulated concentration-response relationships for constant exposure (Figures 1, S1-S3, panels B,H) were constructed by predicting survival over time for a series of exposure levels (see table S13 for details). Predictions were done both in a deterministic (using the optimal parameter vector) and a probabilistic manner (with 10000 Monte Carlo runs for CYP, CBZ and MAL, and 5000 MC runs for DIM, respectively). Simulated survival at day 4 as appearing from the optimal solutions, and median, minimum and maximum as appearing from the probabilistic simulations together with the exposure concentration generated a series of data points which were interpolated to obtain the concentration-response curves.

Concentration-response curves for pulsed exposures

Simulated concentration-response relationships for pulsed exposure scenarios A and B (Figure 3, Figures S1-S4 panels D, F, J, L) were constructed by predicting survival over time for a series of manipulated pulsed exposure profiles. The concentrations as used in the original pulsed exposure experiments A and B were multiplied by a series of multiplication factors (see table S13 for details). Predictions were done both in a deterministic (using the optimal parameter vector) and a probabilistic manner (with 10000 Monte Carlo runs for cypermethrin, carbendazim and malathion, and 5000 MC runs for dimethoate, respectively). Simulated survival at the end of the pulsed experiments as appearing from the optimal parameter sets, and median, minimum and maximum as appearing from the probabilistic simulations together with the exposure concentrations generated a series of data points that were interpolated to create the concentration-response curves at the last day of the experiment.

Table S13: Concentrations used for creating simulated concentration-response relationships.

		Acute	Pulse A & B
Compound	# concentration levels	Range (nmol L^{-1})	Factors (-)[a]
Cypermethrin	29	0.00499 – 5.474	0.0388 – 42.52
Malathion	16	0.223 – 7.390	0.055 – 1.84
Dimethoate	32	0.449 – 812.41	0.010 – 18.08
Carbendazim	42	2.45 – 54176	0.00035 – 7.818

a: factors being multiplied with the original concentration time series as measured in pulse A and B experiments.

Best fit parameters

Table S14: Estimated optimal parameter values, negative log-likelihood values and confidence limits for GUTS-SIC-IT and GUTS-SIC-SD for *G. pulex*

SD model		h_B	k_e	*kk*	*z*	*-ln L*	*Parameter sets*[b]
	Lcl[a]		*0.20284*	*13.212*	*1.348E-2*		
Cypermethrin	opt[a]	*0.0278*	*0.41708*	*29.826*	*2.395E-2*	*85.4141*	*1028/8047*
	ucl[a]		*0.62393*	*91.450*	*3.421E-2*		
	lcl		*4.621E-3*	*1.4398*	*1.721E-2*		
Malathion	opt	*0.0129*	*2.153E-2*	*10.111*	*4.115E-2*	*117.587*	*2201/13828*
	ucl		*3.245E-2*	*312.39*	*1.154E-1*		
	lcl		*1.262E-3*	*3.51E-02*	*1.798E-2*		
Dimethoate	opt	*0.0267*	*1.934E-2*	*4.60E-01*	*1.958E-1*	*145.669*	*1785/10959*
	ucl		*9.909E-2*	*9.35E-01*	*6.935E-1*		
	lcl		*1.069E-4*	-	-		
Carbendazim	opt		*5.819E-4*	*0.5641*	*0*	*195.276*	*3373/14247*
	ucl		-	*29.027*	-		
IT model		h_B	k_e	*alpha*	*beta*	*-ln L*	*Parameter sets*
	lcl		*8.960E-5*	*3.106E-5*	*2.6310*		
Cypermethrin	opt	*0.0278*	*4.008E-4*	*8.611E-5*	*3.7959*	*85.9546*	*1119/7408*
	ucl		*3.201E-2*	*2.012E-4*	*5.1715*		
	lcl		*5.249E-5*	*6.523E-4*	*3.2716*		
Malathion	opt	*0.0129*	*1.255E-4*	*7.228E-4*	*4.2281*	*126.9*	*1979/14277*
	ucl		*8.135E-4*	*7.969E-4*	*5.1392*		
	lcl		*3.724E-5*	*3.186E-3*	*1.6983*		
Dimethoate	opt	*0.0437*	*6.275E-5*	*6.870E-3*	*2.2566*	*165.605*	*780/5418*
	ucl		*2.193E-4*	*2.124E-2*	*2.9299*		
	lcl		-	*9.983E-3*	*1.1849*		
Carbendazim	opt	*0.0564*	*1.006E-5*	*1.722E-2*	*1.9185*	*204.674*	*2053/12231*
	ucl		-	*6.989E-2*	*2.9934*		

[a]*lcl*: lower confidence limit (critical value from Chi-square distribution 3.84, $df = 1$, $\alpha = 0.05$); *ucl*: upper confidence limit ($\alpha = 0.05$); *opt*: optimal parameter value.

[b]*Parameter sets*: number of parameter sets within the joint confidence region (critical value from Chi-square distribution 7.81, $df = 3$, $\alpha = 0.05$)/number of tested parameter sets.

Performance statistics for model predictions

Table S15: Chi^2 values of the predictions of survival under pulsed exposure regimes as measures of model performance.

		Optimal parameters		Medians of probabilistic predictions			
		A	B	A	B	average	Chi^2 limit
**Cypermethrin**	**SD**	118.52	59.72	118.497	51.469	87.051	12.5916
	**IT**	165.241	30.662	162.81	30.186	97.224	
**Malathio**	**SD**	14325	15501	14992	16020	15209	31.4104
	**IT**	813.30	831.31	831.19	837.20	828.25	
**Dimethoate**	**SD**	736.84	19.757	584.86	13.2681	338.68	12.5916
	**IT**	64.853	172.08	69.436	179.75	121.53	
**Carbendazim**	**SD**	2056.5	2399.6	2029.2	2628.5	2278.4	12.5916
	**IT**	1792.4	1566.2	1888.7	1659.6	1726.7	

Additional figures of survival predictions across different exposure patterns

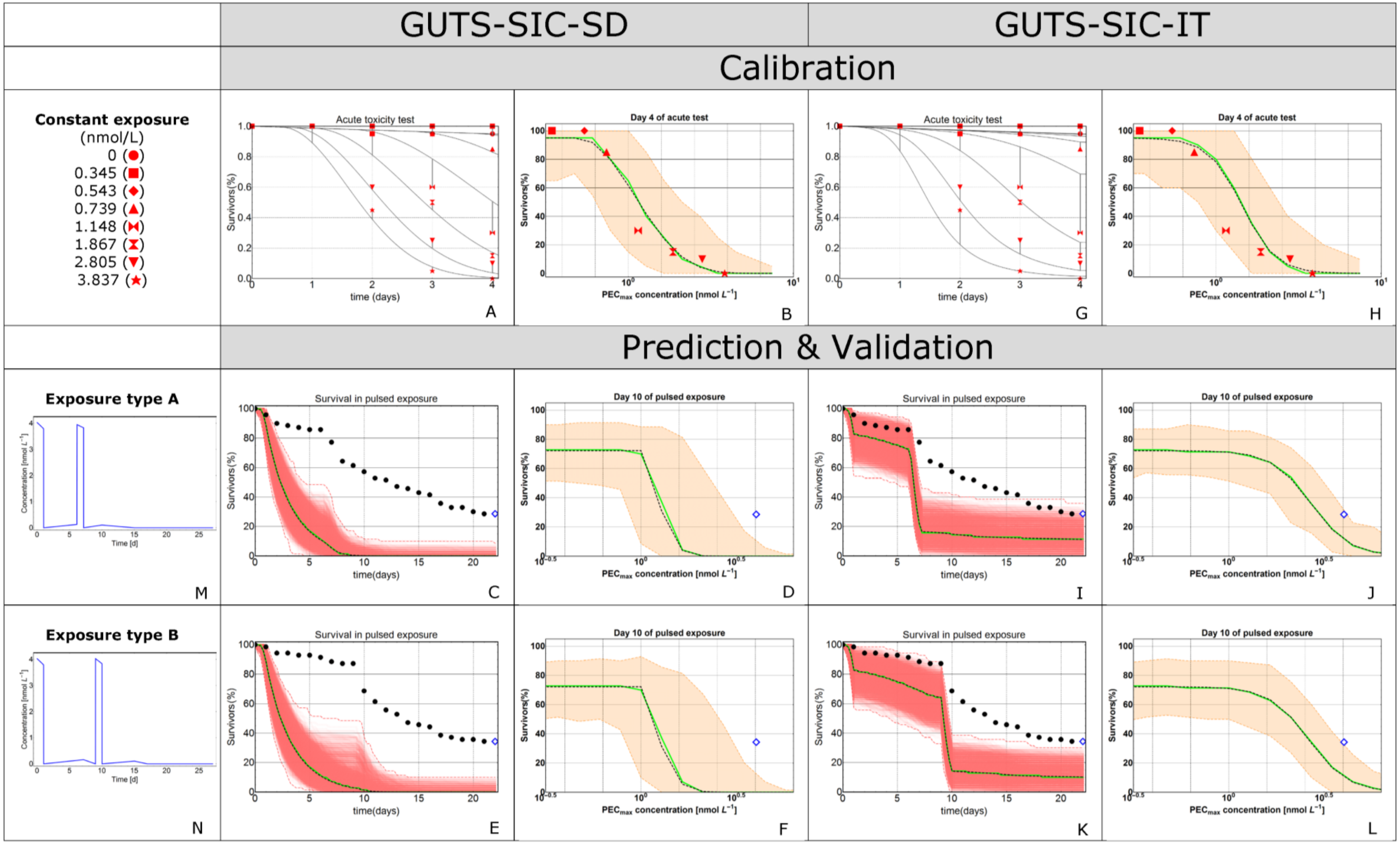

Figure S1: Model calibration and predictions of GUTS-SIC-SD (panels A-F) and GUTS-SIC-IT (panels G-L) for malathion. Calibrated GUTS-SIC-SD (A) and GUTS-SIC-IT (G) survival over time (solid lines) and observed survival (symbols: different treatments). Observed (symbols) and simulated survival (black dashed lines: predictions for the optimal parameter sets) at day 4 of the acute tests in concentration-response plots for GUTS-SIC-SD (B) and GUTS-SIC-IT (H). The shaded areas (red or orange) indicate the confidence regions (95% parametric uncertainty, 100% stochasticity, 10000 simulations), while the solid green line is the median of these predictions. The more intense the red, the more predictions are overlapping. Panels C and I show the observed and simulated survival over time for the exposure type A, as shown in the left column (M). The last data points in panels C, E, I and K are those shown in panels D, F, J and L, indicating observed survival at day 22 of the pulsed exposure. Panels E and K show survival over time for exposure type B (N); panels F and L survival at day 22 of these experiments.

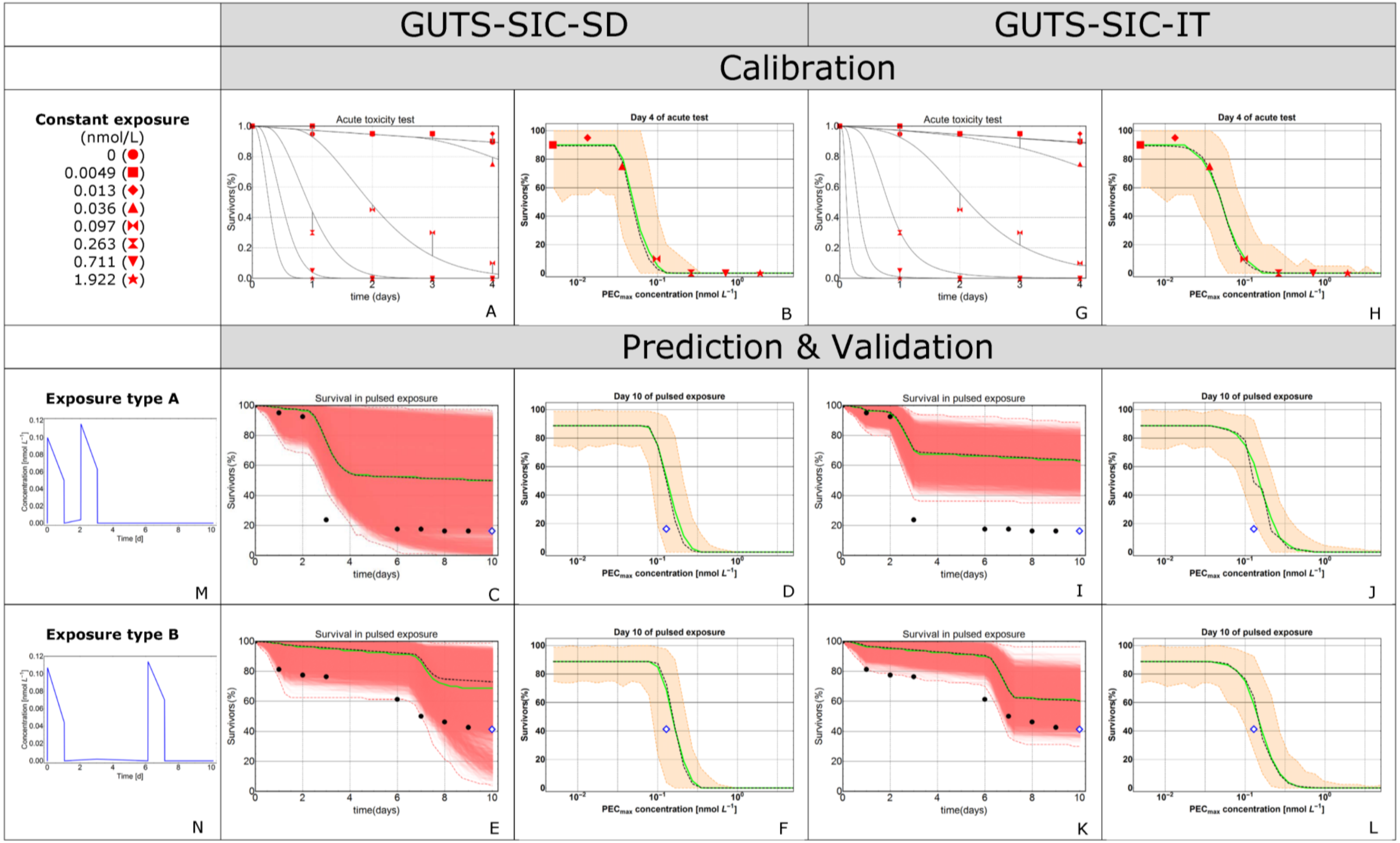

Figure S2: Model calibration and predictions of GUTS-SIC-SD (panels A-F) and GUTS-SIC-IT (panels G-L) for cypermethrin. Calibrated GUTS-SIC-SD (A) and GUTS-SIC-IT (G) survival over time (solid lines) and observed survival (symbols: different treatments). Observed (symbols) and simulated survival (black dashed lines: predictions for the optimal parameter sets) at day 4 of the acute tests in concentration-response plots for GUTS-SIC-SD (B) and GUTS-SIC-IT (H). The shaded areas (red or orange) indicate the confidence regions (95% parametric uncertainty, 100% stochasticity, 10000 simulations), while the solid green line is the median of these predictions. The more intense the red, the more predictions are overlapping. Panels C and I show the observed and simulated survival over time for the exposure type A, as shown in the left column (M). The last data points in panels C, E, I and K are those shown in panels D, F, J and L, indicating observed survival at day 22 of the pulsed exposure. Panels E and K show survival over time for exposure type B (N); panels F and L survival at day 22 of these experiments.

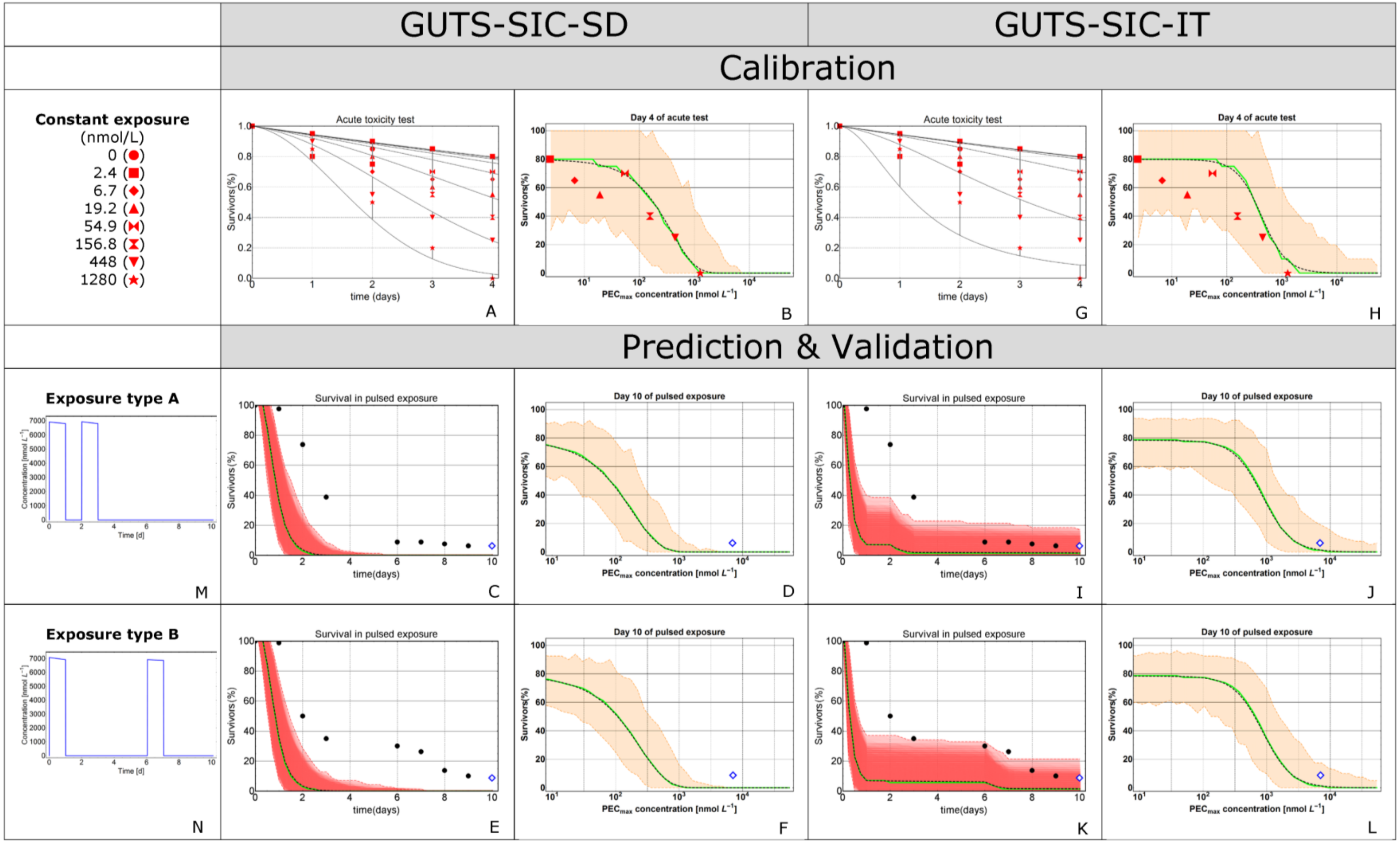

Figure S3: Model calibration and predictions of GUTS-SIC-SD (panels A-F) and GUTS-SIC-IT (panels G-L) for carbendazim. Calibrated GUTS-SIC-SD (A) and GUTS-SIC-IT (G) survival over time (solid lines) and observed survival (symbols: different treatments). Observed (symbols) and simulated survival (black dashed lines: predictions for the optimal parameter sets) at day 4 of the acute tests in concentration-response plots for GUTS-SIC-SD (B) and GUTS-SIC-IT (H). The shaded areas (red or orange) indicate the confidence regions (95% parametric uncertainty, 100% stochasticity, 10000 simulations), while the solid green line is the median of these predictions. The more intense the red, the more predictions are overlapping. Panels C and I show the observed and simulated survival over time for the exposure type A, as shown in the left column (M). The last data points in panels C, E, I and K are those shown in panels D, F, J and L, indicating observed survival at day 22 of the pulsed exposure. Panels E and K show survival over time for exposure type B (N); panels F and L survival at day 22 of these experiments.

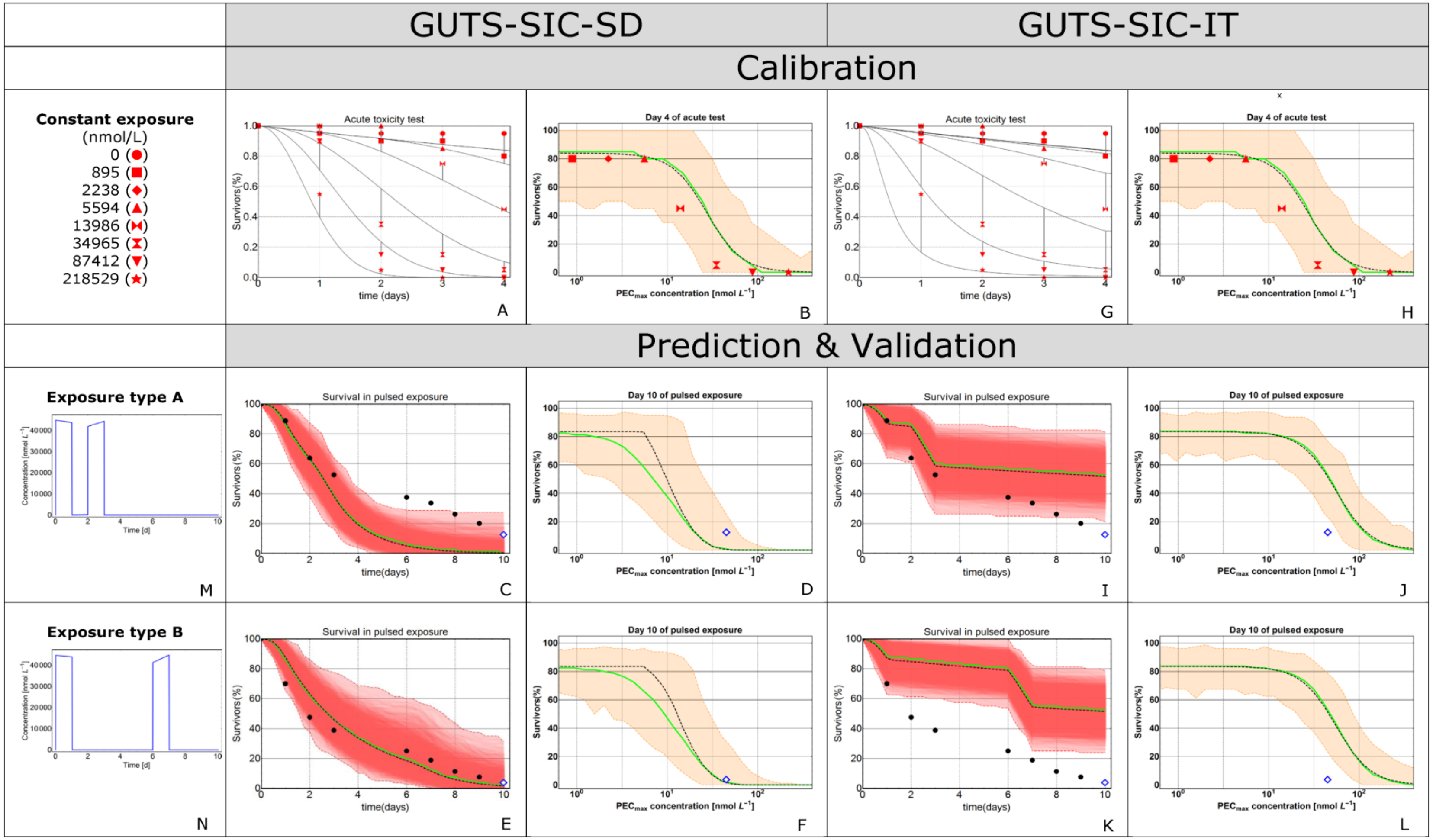

Figure S4: Model calibration and predictions of GUTS-SIC-SD (panels A-F) and GUTS-SIC-IT (panels G-L) for dimethoate. Calibrated GUTS-SIC-SD (A) and GUTS-SIC-IT (G) survival over time (solid lines) and observed survival (symbols: different treatments). Observed (symbols) and simulated survival (black dashed lines: predictions for the optimal parameter sets) at day 4 of the acute tests in concentration-response plots for GUTS-SIC-SD (B) and GUTS-SIC-IT (H). The shaded areas (red or orange) indicate the confidence regions (95% parametric uncertainty, 100% stochasticity, 10000 simulations), while the solid green line is the median of these predictions. The more intense the red, the more predictions are overlapping. Panels C and I show the observed and simulated survival over time for the exposure type A, as shown in the left column (M). The last data points in panels C, E, I and K are those shown in panels D, F, J and L, indicating observed survival at day 22 of the pulsed exposure. Panels E and K show survival over time for exposure type B (N); panels F and L survival at day 22 of these experiments

Detailed assessment of survival predictions across exposure patterns

Background

Exposure concentrations, e.g. in water bodies adjacent to agricultural fields or downstream of sewage outlets and wastewater treatment plants, show highly variable dynamics [16], which mainly depend on chemical application schemes and entry pathways (e.g. spray drift, surface runoff and erosion, or drainage [17]) as well as hydrology and weather [18]. Standard toxicity tests are designed to ensure constant exposure to the chemical over a short test duration and nominal, initially measured, or mean measured concentrations are traditionally used to derive effect thresholds. However, field exposure patterns often differ from the laboratory situation and require assessing the consequences of time-variable exposure patterns on the effect. Toxicokinetic-toxicodynamic models[19] such as GUTS provide a tool to overcome such limitations, i.e. different temporal pattern and overall duration of exposure in toxicity tests and in the field. First, the toxicokinetic-toxicodynamic model is calibrated to the toxicity test data where the nominal, initially measured or mean measured concentrations generally serve as model driving variable (input), and the observed effects are the simulated variable (output). The actual exposure pattern should be represented as accurately as possible, preferably by measured concentration time series[20]. Then the toxicokinetic-toxicodynamic model is run with the environmentally relevant exposure profile as model driving variable (input) to predict effects in the field (output)[9,10].

Calibration

Estimated optimal parameter values, likelihood values and confidence limits are provided in the SI (Table S14) together with values for the model performance indicator Chi^2 (Table S15). Calibration data (Figures S1-S4, upper panel) showed different concentration-response patterns for the various chemicals, which were all captured by the SD and IT model fits. For cypermethrin, the concentration-response curve was relatively steep (Figure S2), and the observed mortality at day 4 was adequately captured by both IT and SD models and optimal parameter values. Parameter uncertainty, as expressed by uncertainty bands of the simulated concentration-response curves, was similar for the SD and the IT model. Negative log-likelihood values were similar for the SD and the IT model, and for the cypermethrin the smallest for all four compounds. AChE inhibitors malathion (Figure S1) and dimethoate (Figure S4) showed shallower concentration-response curve compared to that of cypermethrin (Figure S2). Both models tended to underestimate mortalities for intermediate concentrations, albeit the SD model gave a better fit to dimethoate data. Uncertainty bands were similar in size as those for cypermethrin, and similar between SD and IT. Model error values indicate a better fit for malathion than dimethoate. For both compounds, the SD model fitted data better than the IT model. For the fungicide carbendazim (Figure S3), survival data displayed an inconsistent response: survival for one of the intermediate tested concentrations was similar to that of the control. Moreover, the concentration-response curve was shallower than for other compounds. These properties of the calibration data set resulted in an increase in parameter uncertainty, as reflected in the large uncertainty bands obtained for the probabilistic simulations of both the IT and SD models. Also the negative log-likelihood values for carbendazim were larger than for the other compounds.

Prediction

For assessing the accuracy of our model predictions of the experimentally observed values, we used Chi^2 values (Table S15).

Differences in terms of accuracy and precision of model predictions of survival for the studied compounds were obvious. Survival curves for cypermethrin substantially differed between the exposure regimes A and B (Figure 2, Figure S2) in the magnitude of effects following the second pulse. In the exposure regime A, the second pulse reduced survival from approximately 90% to 20%, whereas in the exposure regime B, the effect of the second pulse was smaller, decreasing survival from approximately 80% to 40%. The difference between A and B can be explained by the time interval between pulses, which may not have been sufficient in regime A to allow for detoxification or recovery from internal damage. In regime B, the time interval between the pulses seemed sufficiently long to ensure recovery. Indeed, the two pulses appeared to have “independent” effects, given that they induced a similar mortality (approximately 20% mortality). Looking at survival over time, it appears that both model predictions based on optimal parameter sets underestimated experimental mortalities but captured the temporal pattern of the response (Figure 2, Figure S2). The medians of the probabilistic simulations were similar to the predictions based on optimal parameters. At the end of the pulsed-exposure experiments, observed survival was always comprised within the uncertainty ranges for the model (except for the IT model under regime A). Partly this is because the uncertainty bands for the SD model were pretty large. Looking at the modelled concentration-response curves, it appears that predictions of the 10d survival with a given model (IT or SD) were similar for both exposure regimes. This can be explained by the fact that the models did not fully reproduce the differences between the pulse regimes (i.e. recovery between pulses).

For both organophosphate insecticides malathion and dimethoate, the survival data were quite similar for both exposure regimes A and B. For dimethoate, a regular decrease in survival was observed over time, which can be interpreted as no effect of the timing of the second pulse. The survival at day 10 was rather similar i.e. 12% and 5% for exposure regimes A and B, respectively. For malathion however, each exposure pulse induced an immediate drop of survival, which occurred at days 1 and 6 in regime A and at days 1 and 10 in regime B. This decrease in survival was more intense after the second peak. Both exposure regimes resulted in survival rates of approximately 30% at day 10. For both compounds, the SD and the IT model differed in the quality of model predictions. The SD model considerably overestimated mortality for both exposure regimes of malathion, but matched the experimental survival over time well for dimethoate (see also Chi-squared values in table S15). The IT model also overestimated mortality for malathion, but to a lesser extent than the SD model. On the other hand, the IT model underestimated mortality for dimethoate. In all cases, the median of probabilistic simulations and the simulations based on optimal parameters were relatively similar. These results are confirmed in the concentration-response view after 10 days.

For the fungicide carbendazim, model predictions based on optimal parameters overestimated mortality over time for both the SD and the IT model and both exposure regimes A and B. The difficulty in calibrating the models based on data leading to a shallow concentration-response curve that showed an inconsistent mortality response at lower concentrations levels explains why probabilistic predictions of the concentration-response relationship exhibited large uncertainty bands. The predicted survival over time showed smaller uncertainty bands presumably because the high mortality pushed the maximum predicted survival to zero relatively soon.

Materials and methods used for the calculation of interspecies variability of effects

Data and model calibration

Survival data for malathion was collected from the literature for five vertebrate species, i.e. *Poecilia reticulata*[21], *Rana sylvatica*[22], *Clarius gariepinus*[23] , *Rana catesbeiana*[24], *Pimephales promelas* [25]. The "scaled internal concentration" option of GUTS, implemented in Matlab, was used to estimate model parameters from individual empirical datasets. Both the individual tolerance model (GUTS-SIC-IT) and the stochastic death model (GUTS-SIC-SD) were fitted. For the individual tolerance (IT) model, a log-logistic median distribution of the threshold was assumed. For model calibration, the Nelder-Mead simplex was applied. For each calibration, the lower value of the dominant rate constant (k_e) was fixed at 0.001 d^{-1} and the upper value was fixed at 10 d^{-1}. This is a realistic range of the dominant rate constant and ensures that the optimization converges within the maximum number of iterations. In addition, the background mortality rate (h_b in d^{-1}) was calibrated on survival data. We used an iterative procedure to find the optimum parameter set for a specific species. Parameterized GUTS-SIC models were used to run virtual concentration-response experiments (using the GUI based version programmed in Delphi) for three exposure scenarios: constant exposure, single pulse exposure at day 1 and double pulse exposure at day 1 and 7. LC50s were estimated at day 14 of the experiments using the drc package[26] in R (R Core Team[3]). HC5 values were determined using MOSAIC_SSD[27].

Best fit parameters for the different species

Table S16: Mean and confidence limits for GUTS-SIC-SD parameter estimates.

Species	k_e (d^{-1})	z ($nmol \cdot L^{-1}$)	k_k ($L \cdot nmol^{-1}$ d^{-1})	h_b (d^{-1})
Rana sylvatica	0.001 [0.001 - 0.543]	1.75 [0 - 828.5	$1.8\ 10^{-3}$ [$3.2\ 10^{-5}$ – $5\ 10^{-3}$]	0.0018 [$1.5\ 10^{-5}$ – 0.013]
Pimephales promelas	1.3 [0.6 - 2.8]	$2.7\ 10^{4}$ [$1.8\ 10^{4}$ – $3.2\ 10^{4}$]	$2.2\ 10^{-5}$ [$1.0\ 10^{-5}$ – $4.0\text{-}10^{-5}$]	0.009 [0.002 - 0.23]
Poecila reticulata	1.11 [0.65 - 3.26]	2130 [1919 - 3372]	0.00022 [0.00012 - 0.00039]	0.0066 [0.00112 - 0.0213]
Rana catesbeiana	1.64 [0.24 - 10]	5375 [2170 - 6321]	$1.1\ 10^{-5}$ [$2.8\ 10^{-6}$ – $2.2\ 10^{-5}$]	0 [0 - 0.001]
Clarias gariepinus	2.1 [1.8 - 2.7]	2065 [1971 - 2102]	7.10^{-5} [5.10^{-5} - 9.10^{-5}]	$1.04\ 10^{-16}$ [0 - 0.03816]

Table S17: Mean and confidence limits for GUTS-SIC-IT parameter estimates.

Species	k_e (d^{-1})	α (nmol)	β (-)	h_b (d^{-1})
Rana sylvatica	0.001 [0.001 – 1.293]	51.23 [32.1 -4692]	2.16 [0.99 – 168.35]	0.0044 [$3.57 \cdot 10^{-5}$ – 0.0178]
Pimephales promelas	0.62 [0.30 - 0.97]	$2.9\ 10^{-4}$ [$2.8\ 10^{-4}$ – $4.7\ 10^{-4}$]	5.9 [3.5 - 9.1]	0.008 [0.001 - 0.22]
Poecila reticulata	0.2291 [0.1322 - 0.3367]	2230 [1599 – 2837]	3.904 [0.442 - 5.515]	7.117e-15 [0 - 0.01728]
Rana catesbeiana	0.12 [0.02 - 0.21]	8105 [4233 - 11170]	4.05 [1.58 - 8.48]	0 [0 - 0.0007]
Clarias gariepinus	0.59 [0.39 - 0.76]	2273 [1962 - 2434]	12.2 [8.1-16.4]	0.026 [0.012 - 0.046]

Survival plots and scaled internal concentrations for the different species

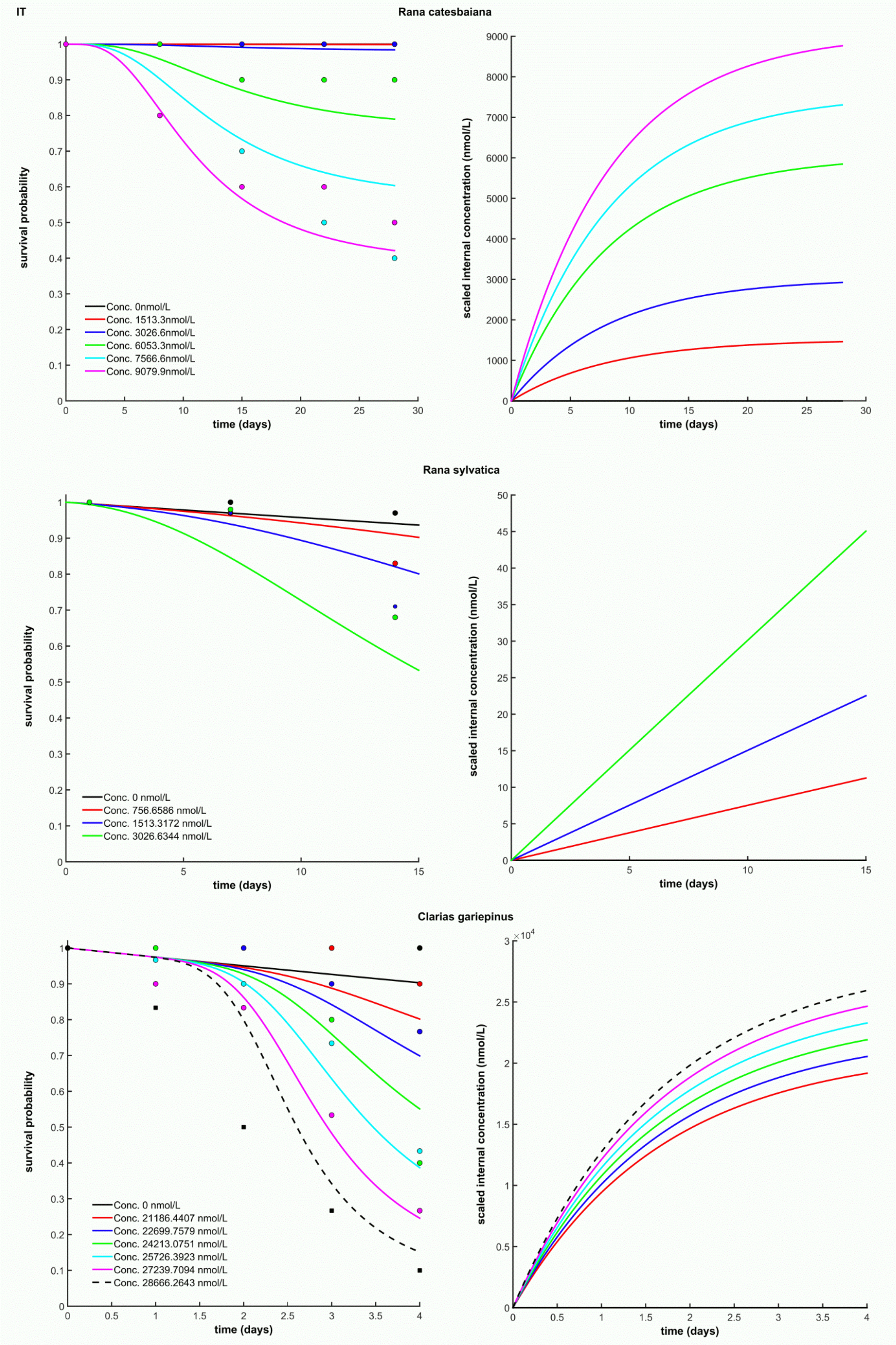

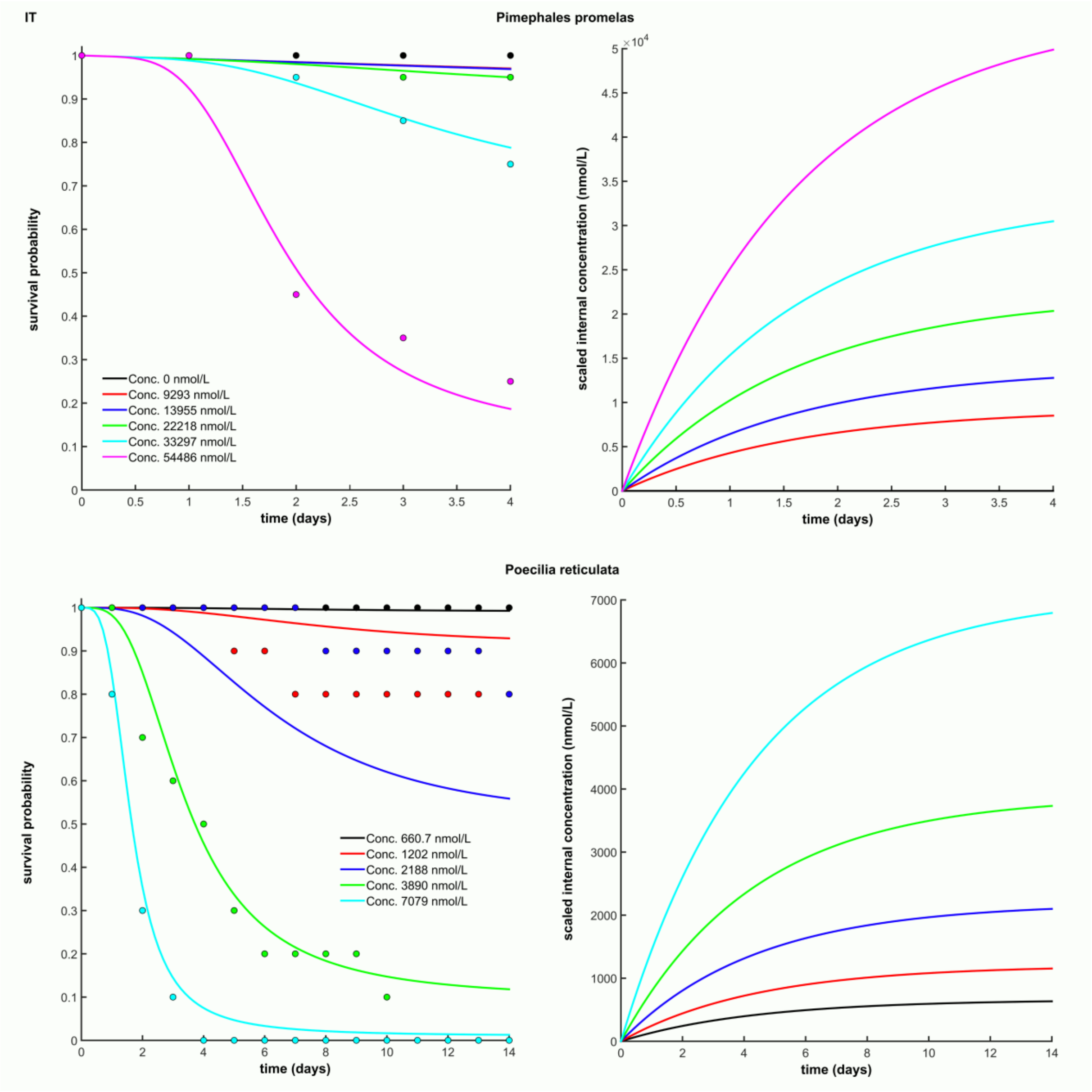

Figure S5: Parameterized GUTS-SIC-IT models for five vertebrate species exposed to malathion. Left panel: Survival as function of time, dots represent data and lines model simulations. Left panel: modelled scaled internal concentration as function of time.

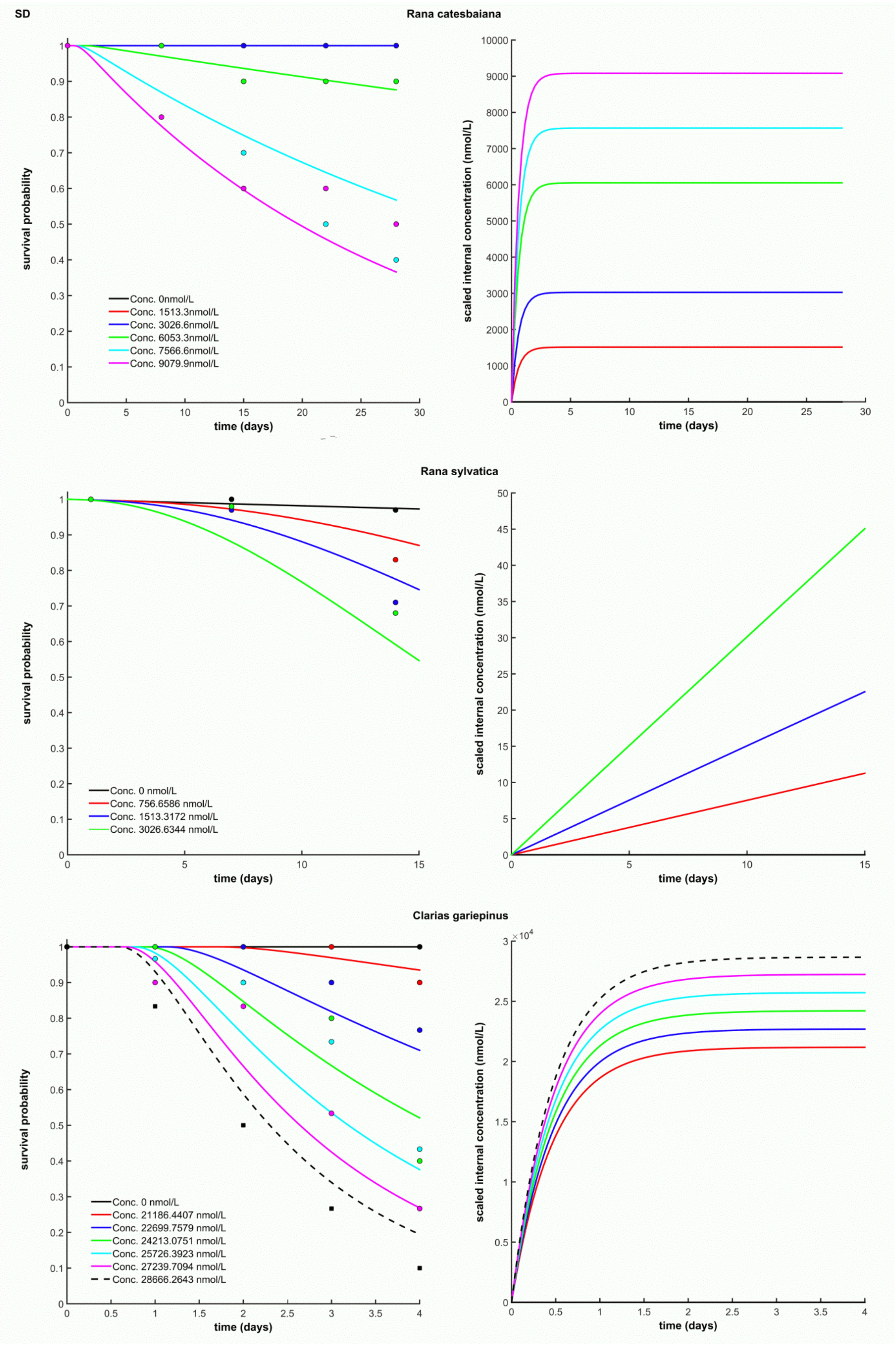
SD
Rana catesbaiana
survival probability
time (days)
scaled internal concentration (nmol/L)
Conc. 0nmol/L
Conc. 1513.3nmol/L
Conc. 3026.6nmol/L
Conc. 6053.3nmol/L
Conc. 7566.6nmol/L
Conc. 9079.9nmol/L
Rana sylvatica
Conc. 0 nmol/L
Conc. 756.6586 nmol/L
Conc. 1513.3172 nmol/L
Conc. 3026.6344 nmol/L
Clarias gariepinus
Conc. 0 nmol/L
Conc. 21186.4407 nmol/L
Conc. 22699.7579 nmol/L
Conc. 24213.0751 nmol/L
Conc. 25726.3923 nmol/L
Conc. 27239.7094 nmol/L
Conc. 28666.2643 nmol/L

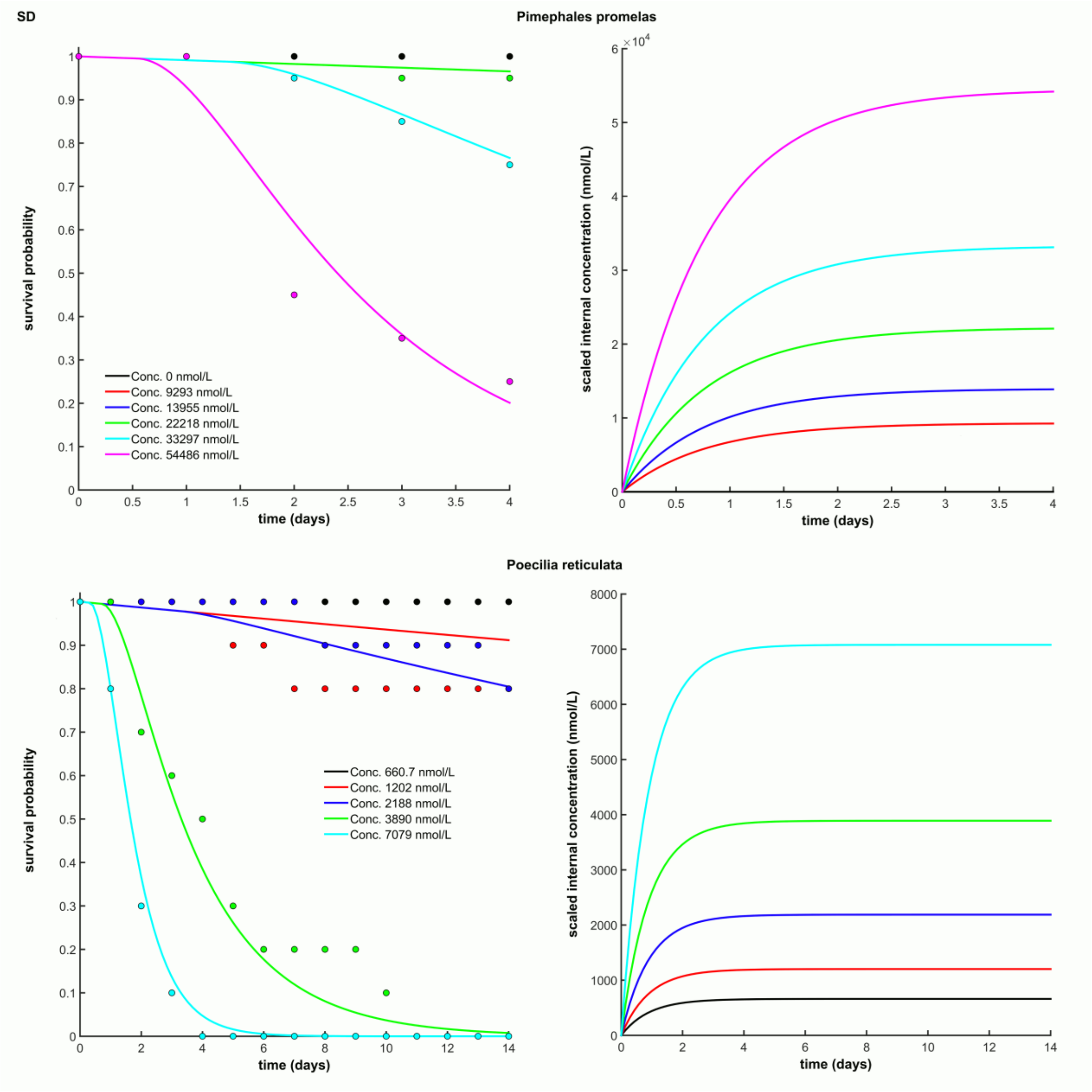

Figure S6: Parameterized GUTS-SIC-SD models for five vertebrate species exposed to malathion. Left panel: Survival as function of time, dots represent data and lines model simulations. Left panel: modelled scaled internal concentration as function of time.

Calculations on synthetic data with Matlab

Calculations were made with the BYOM framework for Matlab and the GUTS2 package. Both can be downloaded free of charge from http://www.debtox.info/byom.html. The GUTS2 package was modified to automate the process of loading datasets from file, fitting them, and writing the results files. To illustrate the fits on the synthetic data sets, two examples are shown in Figure S9. The

Matlab package allows to profile the likelihood function, and in that way create confidence intervals on the model parameters. The overall results for each case and each parameter are shown in Figure S6. The Matlab results are very similar to the R results. Differences in the confidence intervals relate to the differences in approach taken in each package (profile likelihood in Matlab, and Bayesian quantiles on the marginal distributions in R).

Table S18: Model parameters used to generate synthetic data sets.

Model parameter	Value
Dominant rate constant (k_e)	1 d^{-1} (fixed)
Median value of the threshold distribution (z)	0.60 nM
Factor sensitivity differences (F_s)	2 (-) (fixed)
Killing rate (k_k)	0.39 nM^{-1} d^{-1}
Background hazard rate (h_b)	0.0035 d^{-1}

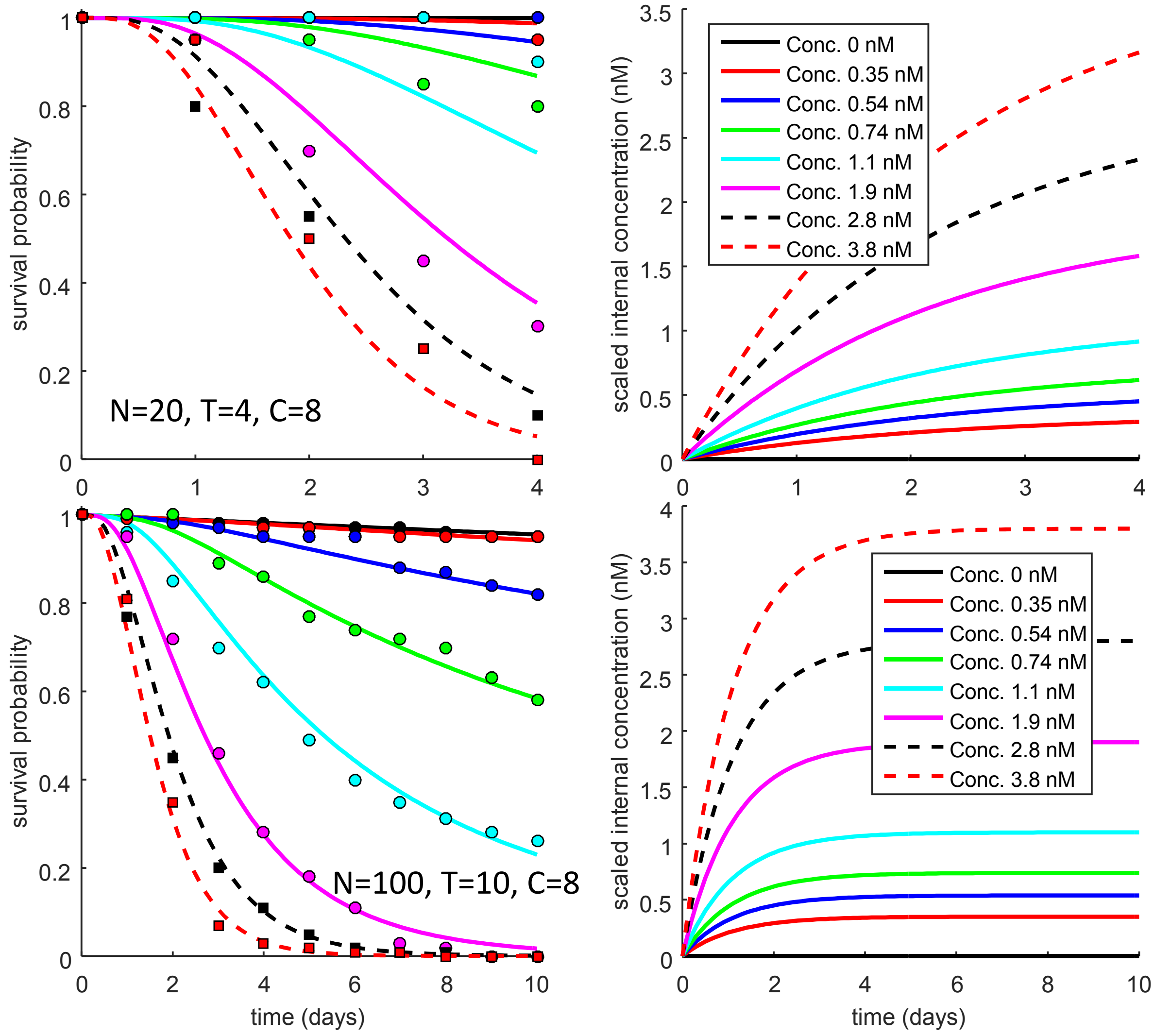

Figure S7: Two example fits with Matlab (best fitting parameter set). Top panels: fit for one replicate data set in Case 1. Bottom panels: fit for one replicate data set in Case 4 (same cases as in Fig. S9). Right panels show the predicted curves for the scaled internal concentration.

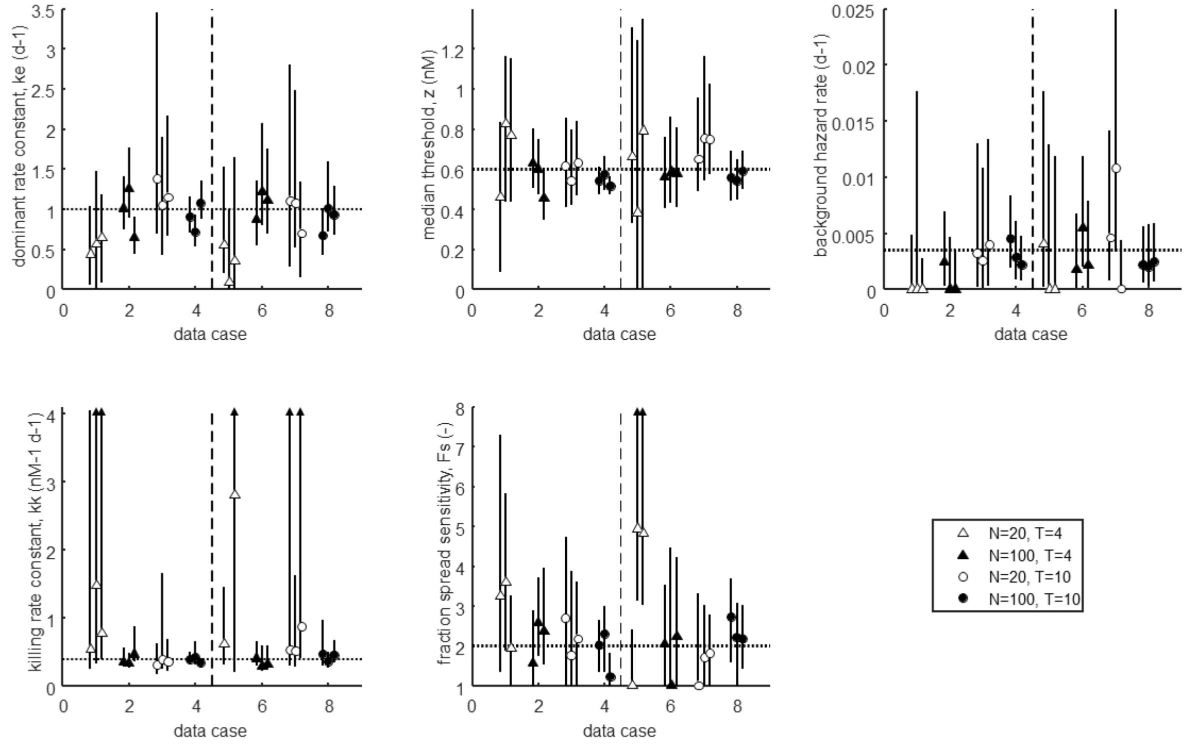

Figure S8: Parameter estimates; best value with 95% confidence intervals. Dotted horizontal line indicates the true parameter value that was used to produce the data sets. Vertical broken line separates the data sets with 8 exposure concentrations (left) from those with only 5 (right). Arrows on confidence intervals indicate that the error bar extends much further (truncated to improve readability).

What is a 'slow' dominant rate constant?

In fitting the different GUTS flavours to the malathion data, it is clear that the dominant rate constant (k_e) cannot be identified from this data set: the survival pattern is best explained by an (almost) linear build-up of the dose metric. This situation of 'slow kinetics' is quite common and leads to a range of problems, as outlined below. For our synthetic data, starting from a situation of slow kinetics is not useful, as we know *a priori* that we will never be able to identify the parameters that we used to generate the data. For that reason, we derived the initial parameter set using a slightly different approach. We started by fixing $k_e = 1$, which provides a situation where we reach 98% of steady state after four days.

If the dominant rate constant (k_e) is slow, this means that the dose metric (e.g., the scaled internal concentration) increases almost linearly in time. This implies that k_e cannot be properly identified from the survival data (it will get a confidence interval that extends to zero), and that the other parameters (k_k and z) can also not be identified (they become heavily correlated to k_e). What is 'slow' depends on the test duration: long tests can identify lower k_e values than short tests. Fig. 1 shows the relationship between test duration and identifiability of k_e. When 90% of steady state is achieved, k_e can be confidently estimated from the data, and even at only 50% of steady state, this is feasible. This implies that in a 4-day test we cannot expect to be able to identify dominant rate constants that are below 0.1 d^{-1}.

This situation of 'slow kinetics' is quite common and leads to a range of problems. Firstly, optimisation routines will not converge on a unique, 'best', set of model parameters as all values of k_e below a certain value can produce the same fit (see Figure S8 for the link between this critical value of k_e and test duration). This results in wide confidence intervals of the type $k_e < x$. Secondly, the other TD model parameters (k_k and z) will become strongly correlated to k_e, and hence will also show wide (half-open) confidence intervals. Several pragmatic strategies are available to deal with this situation:

1) Accept it as is. It is still possible to get a sample from the posterior distribution in this situation, which includes the correlations between the parameters. The model predictions using this sample do not necessarily suffer from the large uncertainties in each model parameter because of these strong correlations.
2) Set an arbitrary minimum value for the elimination rate, based on practical consideration (e.g., the duration of the experiment or the life expectancy of the species). Depending on the purpose of the analysis, such arbitrariness may be acceptable.
3) Use an informed prior to constrain the optimisation. For example, using information from related compounds or from body residues. This latter strategy should be used with care, as k_e does not necessarily relate to whole-body internal concentrations.
4) Reformulate the model to new parameters that *can* be estimated in this situation. This strategy was used in the original DEBtox software[28]. The number of parameters was reduced by one by taking the quotient z/k_e and the product $k_e k_k$ as new parameters. The disadvantage is, however, that these new parameters are impossible to interpret ecotoxicologically.

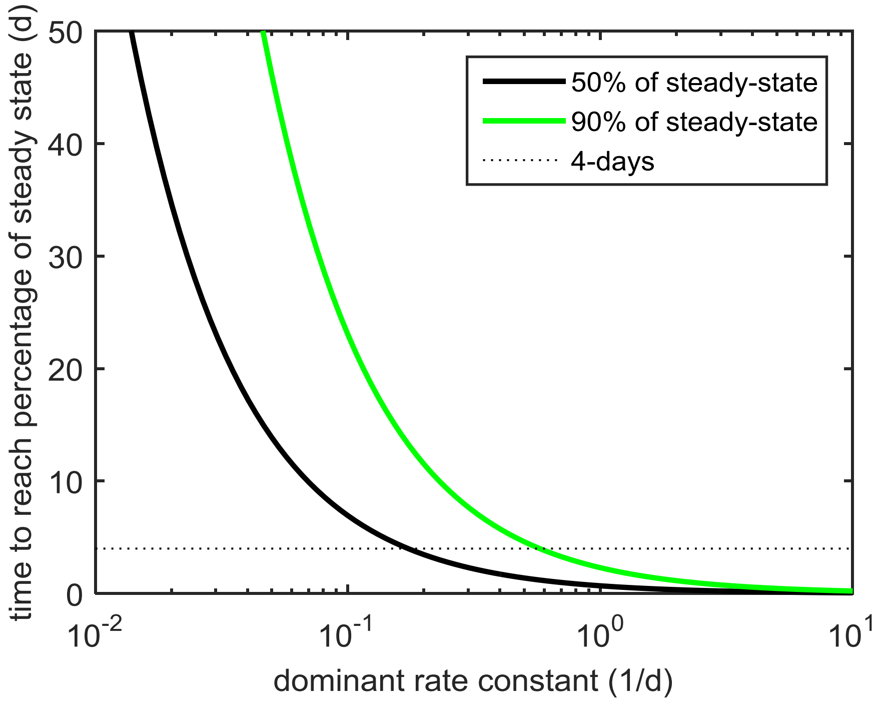

Figure S9: Relationship between the dominant rate constant and the exposure duration needed to obtain a certain percentage of steady state.

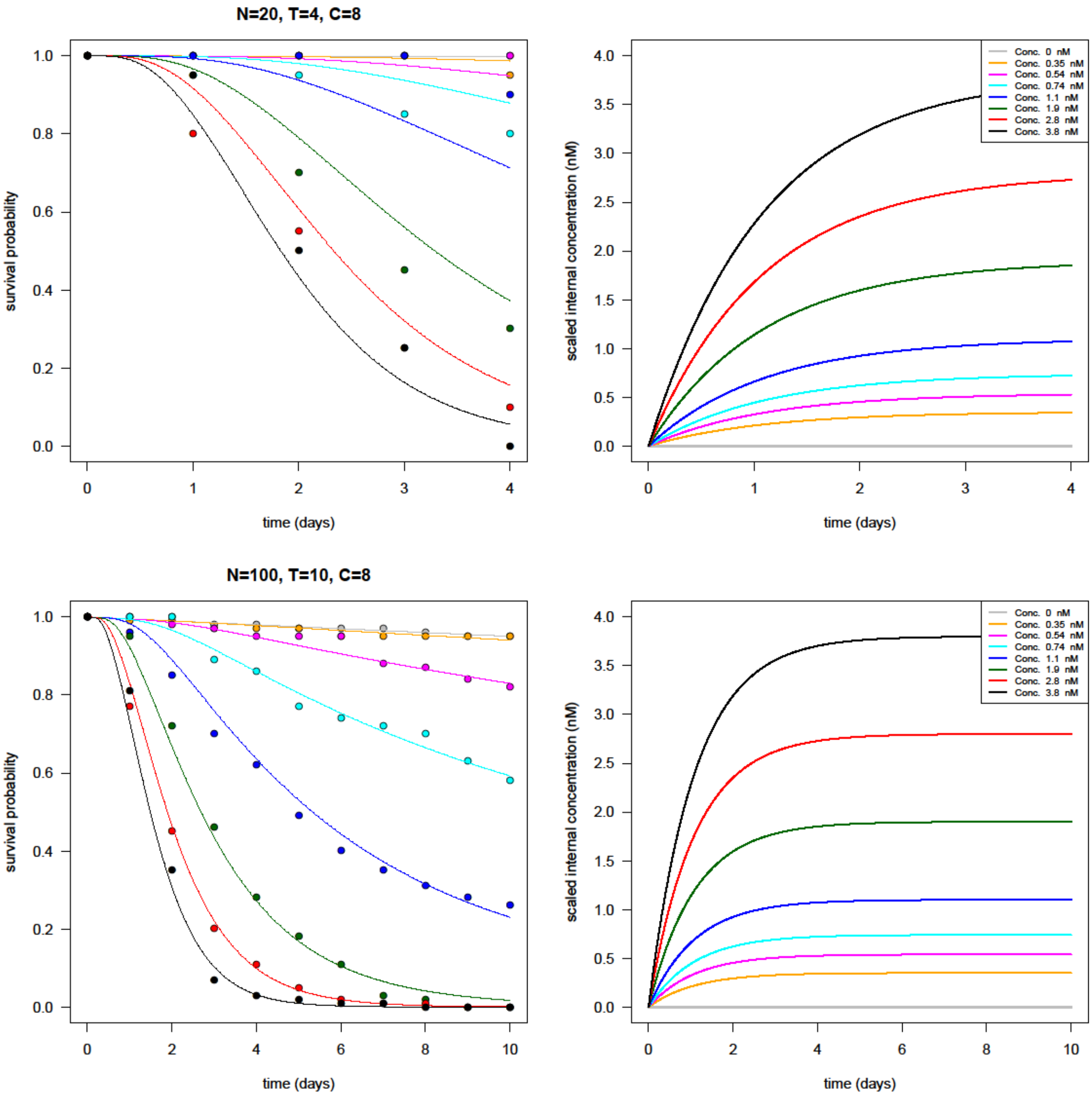

Figure S10: Two example fits with R (best fitting parameter set). Top panels: fit for one replicate data set in Case 1. Bottom panels: fit for one replicate data set in Case 4 (same cases as in Fig. S6). Right panels show the predicted curves for the scaled internal concentration.

References

1 Jager, T., Albert, C., Preuss, T. G. & Ashauer, R. General Unified Threshold Model of Survival - a Toxicokinetic-Toxicodynamic Framework for Ecotoxicology. *Environ. Sci. Technol.* **45**, 2529-2540 (2011).

2 Meeker, W. Q. & Escobar, L. A. Teaching about approximate confidence-regions based on maximum-likelihood-estimation. *Am. Stat.* **49**, 48-53 (1995).

3 R: A language and environment for statistical computing v. http://www.R-project.org (R Foundation for Statistical Computing, Vienna, Austria, 2015).

4 GUTS: fast calculation of the likelihood of a stochastic survival model. R package. v. Version 1.0 (http: //CRAN.R-project.org/package=GUTS, 2015).

5 Gergs, A., Zenker, A., Grimm, V. & Preuss, T. G. Chemical and natural stressors combined: From cryptic effects to population extinction. *Scientific Reports* **3**, doi:10.1038/srep02036 (2013).

6 Kulkarni, D., Daniels, B. & Preuss, T. G. Life-stage-dependent sensitivity of the cyclopoid copepod Mesocyclops leuckarti to triphenyltin. *Chemosphere* **92**, 1145-1153, doi:http://dx.doi.org/10.1016/j.chemosphere.2013.01.076 (2013).

7 Gabsi, F., Hammers-Wirtz, M., Grimm, V., Schäffer, A. & Preuss, T. G. Coupling different mechanistic effect models for capturing individual- and population-level effects of chemicals: Lessons from a case where standard risk assessment failed. *Ecol. Model.* **280**, 18-29, doi:http://dx.doi.org/10.1016/j.ecolmodel.2013.06.018 (2014).

8 Gergs, A., Kulkarni, D. & Preuss, T. G. Body size-dependent toxicokinetics and toxicodynamics could explain intra- and interspecies variability in sensitivity. *Environ. Pollut.* **206**, 449-455, doi:10.1016/j.envpol.2015.07.045 (2015).

9 Ashauer, R., Thorbek, P., Warinton, J. S., Wheeler, J. R. & Maund, S. A method to predict and understand fish survival under dynamic chemical stress using standard ecotoxicity data. *Environ. Toxicol. Chem.* **23**, 954–965 (2013).

10 Nyman, A.-M., Schirmer, K. & Ashauer, R. Toxicokinetic-toxicodynamic modelling of survival of *Gammarus pulex* in multiple pulse exposures to propiconazole: model assumptions, calibration data requirements and predictive power. *Ecotoxicology* **21**, 1828-1840 (2012).

11 Ashauer, R., O'Connor, I., Hintermeister, A. & Escher, B. I. Death Dilemma and Organism Recovery in Ecotoxicology. *Environ. Sci. Technol.* **49**, 10136-10146, doi:10.1021/acs.est.5b03079 (2015).

12 Vugrin, K. W., Swiler, L. P., Roberts, R. M., Stucky-Mack, N. J. & Sullivan, S. P. Confidence region estimation techniques for nonlinear regression in groundwater flow: Three case studies. *Water Resour. Res.* **43**, doi:10.1029/2005wr004804 (2007).

13 Kon Kam King, G., Delignette-Muller, M. L., Kefford, B. J., Piscart, C. & Charles, S. Constructing Time-Resolved Species Sensitivity Distributions Using a Hierarchical Toxico-Dynamic Model. *Environ. Sci. Technol.* **49**, 12465-12473, doi:10.1021/acs.est.5b02142 (2015).

14 Ashauer, R., Hintermeister, A., Caravatti, I., Kretschmann, A. & Escher, B. I. Toxicokinetic-toxicodynamic modeling explains carry-over toxicity from exposure to diazinon by slow organism recovery. *Environ. Sci. Technol.* **44**, 3963-3971 (2010).

15 Ashauer, R., Hintermeister, A., Potthoff, E. & Escher, B. I. Acute toxicity of organic chemicals to *Gammarus pulex* correlates with sensitivity of *Daphnia magna* across most modes of action. *Aquat. Toxicol.* **103**, 38-45 (2011).

16 Wittmer, I. K. *et al.* Significance of urban and agricultural land use for biocide and pesticide dynamics in surface waters. *Water Res.* **44**, 2850-2862 (2010).

17 FOCUS. FOCUS Surface Water Scenarios in the EU Evaluation Process under 91/414/EEC. 1-238 (European Commission, Health & Consumer Protection Diretorate-General, Brussel, Belgium, 2001).

18 Keller, V. D. J., Williams, R. J., Lofthouse, C. & Johnson, A. C. WORLDWIDE ESTIMATION OF RIVER CONCENTRATIONS OF ANY CHEMICAL ORIGINATING FROM SEWAGE-TREATMENT PLANTS USING DILUTION FACTORS. *Environ. Toxicol. Chem.* **33**, 447-452, doi:10.1002/etc.2441 (2014).
19 Ashauer, R. & Escher , B. I. Advantages of toxicokinetic and toxicodynamic modelling in aquatic ecotoxicology and risk assessment. *J. Environ. Monit.* **12**, 2056 - 2061 (2010).
20 Jager, T. Reconsidering sufficient and optimal test design in acute toxicity testing. *Ecotoxicology* **23**, 38-44 (2014).
21 De Bruijn, J. & Hermens, J. Qualitative and quantitative modelling of toxic effects of organophosphorous compounds to fish. *Sci. Total Environ.* **109–110**, 441-455 (1991).
22 Krishnamurthy, S. V. & Smith, G. R. Combined effects of malathion and nitrate on early growth, abnormalities, and mortality of wood frog (Rana sylvatica) tadpoles. *Ecotoxicology* **20**, 1361-1367, doi:10.1007/s10646-011-0692-3 (2011).
23 Ahmad, Z. Toxicity bioassay and effects of sub-lethal exposure of malathion on biochemical composition and haematological parameters of *Clarias gariepinus*. *African Journal of Biotechnology* **11**, 8578-8585 (2012).
24 Fordham, C. L., Tessari, J. D., Ramsdell, H. S. & Keefe, T. J. Effects of malathion on survival, growth, development, and equilibrium posture of bullfrog tadpoles (Rana catesbeiana). *Environ. Toxicol. Chem.* **20**, 179-184 (2001).
25 Geiger, D. L., Call, D. J. & Brooke, L. T. Acute toxicities of organic chemicals to fathead minnow (Pimephales promelas). Volume IV., (University of Wisconsin-Superior, Superior, Wisconsin, USA, 1988).
26 Ritz, C. & Streibig, J. C. Bioassay analysis using R. *Journal of Statistical Software* **12**, 1-22 (2005).
27 Kon Kam King, G., Veber, P., Charles, S. & Delignette-Muller, M. L. MOSAIC_SSD: A new web tool for species sensitivity distribution to include censored data by maximum likelihood. *Environ. Toxicol. Chem.* **33**, 2133-2139, doi:10.1002/etc.2644 (2014).
28 Kooijman, S. A. L. M. & Bedaux, J. J. M. 150 (VU University Press, Amsterdam, 1996).
